# Intermittent fasting reprograms the brain proteome to prevent synaptic degeneration and cognitive impairment in vascular dementia

**DOI:** 10.7150/thno.119422

**Published:** 2025-07-25

**Authors:** Nishat I. Tabassum, Sharmelee Selvaraji, Yibo Fan, Vernise JT. Lim, Xiangru Cheng, Xiangyuan Peng, Aayushi Arora, Vismitha Rajeev, Julian Ratcliffe, Chad J. Johnson, Keshava K. Datta, Rohan Lowe, Mansour Ebrahimi, Quynh Nhu Dinh, T Michael De Silva, Christopher G. Sobey, Peiyan Wong, Eddie Feng-Ju Weng, Dong-Gyu Jo, Christopher P. Chen, Mitchell K.P. Lai, Thiruma V. Arumugam

**Affiliations:** 1 Department of Microbiology, Anatomy, Physiology and Pharmacology, School of Agriculture, Biomedicine and Environment, La Trobe University, Melbourne, Australia.; 2 La Trobe Institute for Molecular Science, La Trobe University, Melbourne, Australia.; 3 Research Laboratory of Electronics, Department of Materials Science and Engineering, Massachusetts Institute of Technology, Cambridge, MA, USA.; 4 McGovern Institute for Brain Research, Massachusetts Institute of Technology, Cambridge, MA, USA.; 5 Memory Aging and Cognition Centre, Department of Pharmacology, Yong Loo Lin School of Medicine, National University of Singapore, Singapore.; 6 Bioimaging Platform, La Trobe University, Bundoora, VIC, Australia.; 7 La Trobe University-Proteomics and Metabolomics Platform (LTU-PMP), La Trobe Institute for Molecular Science, La Trobe University, Melbourne, Australia.; 8 Centre for Cardiovascular Biology and Disease Research, La Trobe Institute for Molecular Sciences, La Trobe University, Bundoora, Victoria, 3086, Australia.; 9 Baker Heart and Diabetes Institute, Melbourne, Victoria, 3004, Australia; 10 Signature Research Programme in Neuroscience & Behavioural Disorders, Duke-NUS Medical School, Singapore.; 11 Neuroscience and Brain Disease Centre, China Medical University, Taichung, Taiwan.; 12 Graduate Institute of Biomedical Sciences, College of Medicine, China Medical University, Taichung, Taiwan.; 13 School of Pharmacy, Sungkyunkwan University, Suwon, Republic of Korea.

**Keywords:** intermittent fasting, vascular dementia, synaptic loss, cognitive impairment, neuronal death

## Abstract

**Rationale:** Vascular dementia (VaD), driven by chronic cerebral hypoperfusion (CCH), leads to synaptic degeneration and cognitive decline, yet mechanisms linking vascular dysfunction to synaptic loss remain unclear. Intermittent fasting (IF) has emerged as a potential intervention, but its effects on synaptic integrity in VaD are unknown. This study aims to investigate the effects of IF against synaptic degeneration and cognitive impairment induced by CCH.

Methods: Bilateral common carotid artery stenosis (BCAS) was employed to induce chronic CCH by placing 0.18 mm micro-coils around each common carotid artery in mice. To assess temporal differences, the coils remained in place for 1, 7, 14, or 30 days. IF was implemented for 16 hours daily over three months prior to BCAS induction. Cognitive impairment was evaluated using the Barnes maze test. White matter lesions (WMLs) and neuronal loss were assessed using Luxol fast blue and cresyl violet staining, respectively. Immunoblotting and immunohistochemistry were performed to quantify synaptic protein levels. Synaptic integrity was examined using transmission electron microscopy. Proteomic analysis of the hippocampus was conducted to investigate molecular adaptations to IF following CCH.

**Results:** We demonstrate that a 16-hour IF regimen preserves cognitive function and synaptic density despite persistent hypoperfusion. Behavioral assays revealed that IF prevented spatial memory deficits in BCAS mice, while electron microscopy confirmed synaptic preservation without altering baseline architecture. Surprisingly, key synaptic protein levels remained unchanged, suggesting IF protects synaptic function rather than abundance. Proteomic profiling revealed dynamic hippocampal adaptations under IF, including upregulation of synaptic stabilizers, enhanced GABAergic signaling, and suppression of neuroinflammatory mediators. CCH induced microglial engulfment of synapses, suggesting a role in complement-mediated synaptic pruning. Temporal pathway analysis revealed IF's multi-phase neuroprotection: early synaptic reinforcement, mid-phase metabolic optimization, and late-phase suppression of chronic neuroinflammation.

**Conclusion:** These findings establish IF as a potent modulator of synaptic resilience in VaD, acting through coordinated preservation of synaptic structure, inhibition of inflammatory synapse loss, and metabolic reprogramming. Our results highlight IF's potential as a non-pharmacological strategy to combat vascular cognitive impairment by targeting the synaptic vulnerability underlying dementia progression.

## Introduction

Vascular dementia (VaD), the second most common form of dementia worldwide, represents a growing public health challenge with limited therapeutic options 1. Unlike Alzheimer's disease (AD), which is characterized by the accumulation of amyloid-β plaques and neurofibrillary tau tangles, VaD arises primarily from cerebrovascular dysfunction, particularly chronic cerebral hypoperfusion (CCH) secondary to large-vessel disease or microangiopathy 2. This sustained reduction in cerebral blood flow initiates a pathological cascade characterized by oxidative stress, neuroinflammation, and blood-brain barrier disruption, culminating in white matter lesions (WMLs), synaptic degeneration, and neuronal loss 3. While these neurovascular changes are well-documented, the precise mechanisms linking CCH to synaptic damage - a critical determinant of cognitive decline across dementia types - remain poorly understood, leaving a significant knowledge gap in developing targeted interventions.

The vulnerability of synapses to vascular insults presents a particularly compelling therapeutic target. In healthy brains, synaptic plasticity depends on the precise coordination of presynaptic proteins like synaptophysin and synapsins that regulate neurotransmitter release, with postsynaptic scaffolds such as PSD-95 that organize receptor complexes 4. Emerging evidence suggests CCH may disrupt this delicate balance through multiple pathways, including microglial-mediated synaptic pruning, excitotoxicity from glutamate dysregulation, and metabolic stress from impaired oxygen delivery 5. Recent studies have identified complement-dependent phagocytosis of synapses by activated microglia as a key mechanism in other neurodegenerative diseases 6, but whether similar processes operate in VaD remains unknown. This gap in understanding is particularly concerning given that synaptic density correlates more strongly with cognitive function than traditional markers like amyloid plaques or white matter lesions.

Intermittent fasting (IF) has emerged as a powerful modulator of brain health with pleiotropic effects that may specifically address VaD pathology 7, 8. Beyond its well-characterized metabolic benefits, IF induces a neuroprotective state through multiple molecular pathways: enhancing mitochondrial resilience via PGC-1α activation 9, reducing oxidative damage through Nrf2 signaling 10, and suppressing neuroinflammation by inhibiting NLRP3 inflammasome activation 11. Perhaps most intriguingly, IF upregulates brain-derived neurotrophic factor (BDNF), a critical mediator of synaptic plasticity, while simultaneously promoting autophagy-mediated clearance of damaged cellular components 12. These effects position IF as a uniquely comprehensive intervention that could potentially interrupt the vicious cycle of neurovascular dysfunction and synaptic degeneration characteristic of VaD.

Our study addresses these possibilities using an integrated experimental approach in the bilateral common carotid artery stenosis (BCAS) mouse model of CCH, which recapitulates key features of human VaD including progressive white matter injury and cognitive impairment 13. Through a combination of behavioral analyses, ultrastructural examination of synaptic integrity, and unbiased proteomic profiling, we demonstrate that IF not only preserves cognitive function but also maintains synaptic density despite ongoing CCH. Mechanistically, we uncover a neuroprotective pathway in which IF restores the expression of key presynaptic vesicle proteins, thereby preserving synaptic integrity. These findings significantly advance our understanding of both VaD pathogenesis and IF's neuroprotective mechanisms, while highlighting synaptic preservation as a viable therapeutic strategy for vascular cognitive impairment. With growing recognition that vascular factors contribute to most dementia cases, including mixed AD/VaD pathology, our findings suggest IF may have broader applications in cognitive aging.

## Methods

### Experimental animals and Intermittent Fasting (IF) regime

10 weeks old male C57BL/6 mice were obtained from the Animal Resource Centre (Perth, Western Australia) and housed in the La Trobe Animal Research and Teaching Facility (LARTF) under a 12-hour light/dark cycle (lights on at 07:00, lights off at 19:00) with ad libitum access to food and water. Up to four mice were housed per individually ventilated cage and provided with unrestricted access to water and a standard diet containing 20% crude protein, 5% crude fat, 6% crude fiber, 0.5% added salt, 0.8% calcium, and 0.45% phosphorus (Ridley, Victoria, Australia). At 12 weeks of age, mice were randomly assigned to either an *ad libitum* (AL) feeding group or an intermittent fasting (IF) group. Mice in the IF group underwent daily fasting for 16 hours (16:00-08:00) for a duration of three months, whereas AL mice had unrestricted access to both food and water. Based on our prior testing of various IF regimens, we selected this 16-hour active-phase fasting protocol (16:00-08:00) for the current study, as previous evidence indicates that this duration of daily fasting is critical for promoting ischemic tolerance through IF 14. At the study endpoint, body weight, blood glucose, and ketone levels were measured. Blood glucose and ketone concentrations were assessed using the FreeStyle Optium Neo device and corresponding FreeStyle Optium test strips (Abbott Laboratories, Illinois, USA). All in vivo experimental procedures were approved by La Trobe University Animal Care and Use Committees (Ethics approval number: AEC21012) and conducted in compliance with the Australian Code for the Care and Use of Animals for Scientific Purposes (8th edition) and the NIH Guide for the Care and Use of Laboratory Animals. Every effort was made to minimize animal suffering and reduce the number of animals used. The study was conducted in accordance with ARRIVE guidelines.

### Bilateral Common Carotid Artery Stenosis (BCAS) mouse model

Following three months IF, mice were anesthetized with isoflurane and subjected to BCAS surgery. Microcoils specifically designed for murine application (piano wire diameter: 0.08 mm, internal diameter: 0.18 mm, coiling pitch: 0.5 mm, total length: 2.5 mm; Sawane Spring Co Ltd, Japan) were used for the procedure. BCAS was performed by sequentially exposing the left and right common carotid arteries (CCAs), carefully freeing them from their sheaths, and rotating a microcoil around each CCA to induce stenosis. Sham surgeries were conducted as controls, where the surgical site was opened, and the CCAs were gently touched with forceps without microcoil insertion. The surgical site was closed using surgical glue, and mice were monitored postoperatively to ensure full recovery and unrestricted access to food and water ad libitum. Euthanasia was performed via carbon dioxide inhalation at designated time points (1, 7, 14, 30, and 45 days post-BCAS) for subsequent analysis.

### Luxol Fast Blue (LFB) and Cresyl Violet staining

Paraformaldehyde-fixed 4 µm paraffin-embedded sections were deparaffinized, rehydrated, and immersed in LFB solution (Abcam, UK) at 37°C overnight. Excess staining was removed using 95% ethanol, followed by washing with deionized water. To differentiate gray and white matter, sections were incubated in 0.05% aqueous lithium carbonate (Abcam, UK) for 20 seconds, followed by treatment with 70% ethanol until nuclear staining was fully decolorized. Next, sections were immersed in Cresyl Violet solution (Abcam, UK) for 5 minutes, washed with deionized water, dehydrated, cleared in xylene, and mounted for microscopy. Slides were scanned using the Zeiss Axio Scanner 7, and white matter lesions were evaluated in the caudoputamen, internal capsule, and corpus callosum (median and paramedian) regions. Lesion severity was graded as follows: Grade 0 (normal), Grade 1 (disarrangement of nerve fibers), Grade 2 (formation of marked vacuoles), and Grade 3 (disappearance of myelinated fibers). Lesion quantification was performed by averaging the grading scores assigned by three blinded examiners.

### Immunoblot analysis

Cerebral cortex tissues were homogenized in lysis buffer (Thermo Fisher Scientific, USA) and supplemented with protease inhibitors (Thermo Fisher Scientific, USA) and phosphatase inhibitors (Thermo Fisher Scientific, USA) to prevent protein degradation and dephosphorylation during extraction. The homogenate was mixed with 2× Laemmli buffer (Bio-Rad Laboratories, USA), and protein samples were subjected to sodium dodecyl sulfate-polyacrylamide gel electrophoresis (SDS-PAGE) (Bio-Rad Laboratories, USA) using Tris-glycine running buffer. Proteins were transferred onto nitrocellulose membranes using a Bio-Rad transfer apparatus in a buffer containing 0.025 mol/L Tris base, 0.15 mol/L glycine, and 10% (v/v) methanol for 1 hour and 40 minutes at 350 mA. Membranes were then incubated overnight at 4°C with agitation in primary antibodies against Synaptophysin (ABclonal Technology, A19122, USA), Synapsin II (ABclonal Technology, A19542, USA), Syntaxin 1A (Cell Signaling Technology, D4E2W, USA), Syntaxin 6 (Cell Signaling Technology, C34B2, USA), Shank 3 (ABclonal Technology, A20488, USA), PSD95 (ABclonal Technology, A0131, USA), Neuroligin 1 (Invitrogen, PA5-52323, USA), and β-actin (Sigma-Aldrich, A5441, USA). After primary antibody incubation, membranes were washed three times with 1× TBST (10-minute washes each) and subsequently incubated for 1 hour at room temperature with a horseradish peroxidase (HRP)-conjugated goat anti-rabbit secondary antibody (ABclonal Technology, AS014, USA) with agitation. Following secondary antibody incubation, membranes underwent three additional 10-minute washes with 1× TBST before enhanced chemiluminescence substrate (Bio-Rad Laboratories, USA) was applied. Protein bands were visualized using the ChemiDocTM MP imaging system (Bio-Rad Laboratories, USA). Protein quantification was performed using ImageJ software (Version 1.54; National Institutes of Health, USA), with densitometry values normalized to corresponding β-actin levels.

### Immunohistochemistry

Both paraffin-embedded brain sections (4 µm) and cryostat sections (14 µm) were used for immunohistochemistry. Cryostat sections and deparaffinized paraffin sections were rehydrated in phosphate-buffered saline (PBS) prior to antigen retrieval, which was performed by boiling the slides in sodium citrate buffer (113.93 mM Na₃C₆H₅O₇, pH 6) for 1 minute. Sections were then rinsed twice in PBS (5-minute and 10-minute washes, respectively) before blocking for 2 hours in a solution containing 3% (v/v) horse serum and 0.5% (v/v) Triton X-100. Following blocking, sections were incubated overnight with primary antibodies targeting PSD95 (Abcam, ab18258, UK), Shank3 (ABclonal Technology, A20488, USA), Synapsin II (ABclonal Technology, A19542, USA), Synaptophysin (ABclonal Technology, A19122, USA), Iba1 (Novus Biologicals, NB100-1028, USA), and MAP2 (Merck, MAB3418, USA). After primary antibody incubation, sections were washed twice in PBS-Tween (0.5% v/v) before incubation with host-specific, fluorophore-conjugated secondary antibodies. Sections were then mounted using ProLong™ Diamond Antifade Mountant with DAPI (Thermo Fisher Scientific, P36962, USA). Super-resolution images were acquired using a Zeiss LSM 900 Confocal Microscope equipped with Airyscan 2 and further annotated using Zen Blue (Zeiss) and ImageJ software.

### Transmission electron microscopy

Mouse cortical tissue was post-fixed in modified Karnovsky's fixative, consisting of 2.5% glutaraldehyde (ProSciTech, C002, Australia), 2% paraformaldehyde (ProSciTech, #C004), and 0.1 M Sorensen's buffer (ProSciTech, ASOR68C, Australia). Tissue processing was performed using the Pelco BioWave (ProSciTech, PEL36700-230, Australia). After rinsing in 0.1 M Sorensen's buffer, tissues were post-fixed with 1% osmium tetroxide (ProSciTech, C011-2, Australia) and 1.5% potassium ferrocyanide (ProSciTech, EMS25154-20, Australia) in 0.1 M Sorensen's buffer, followed by additional rinsing in distilled water. Dehydration was performed using a graded ethanol series (30%, 50%, 70%, 90%, 95%, and 100%), and tissues were further rinsed in 100% acetone before embedding in Spurr's resin. Ultrathin sections (~70 nm) were prepared using a Leica UC7 Ultramicrotome (Leica Microsystems Pty Ltd, Australia) and stained with 2.5% uranyl acetate (ProSciTech, C079, Australia) and lead citrate (ProSciTech, C073, Australia). Images were acquired in a blinded manner using a JEOL 2100 Transmission Electron Microscope (Akishima, Japan) at 80 kV and 10,000× magnification. Synaptic counting was performed using size-frequency analysis in ImageJ 15, with post-synaptic density length and G-ratio also measured using ImageJ software. The G-ratio was determined as the ratio of the inner to outer diameter of the axon.

### Barnes maze test

The Barnes maze test was conducted on 6-month-old male C57BL/6 mice, three weeks after BCAS surgery. The test was performed on an elevated circular platform (1 meter in diameter) with 20 equally spaced holes along the perimeter, with an escape box placed beneath one of the holes. Visual-spatial cues were positioned around the maze and remained consistent throughout the study. Prior to testing, mice were acclimated to the experimental room for at least 1 hour. During the acquisition phase, on Day 1, each mouse was first placed inside the escape box for 1 minute, then positioned at the center of the maze under a black chamber. After 10 seconds, the chamber was lifted, and an aversive stimulus (light ~500 lux and buzzer at 85-90 dB) was activated. Mice were allowed 3 minutes to explore the maze or until they entered the escape box. Once inside, the stimulus was turned off, and the escape hole was covered for 30 seconds. This procedure was repeated once daily for four consecutive days (excluding placement inside the escape box). The latency to locate the target hole was recorded, and the platform was cleaned with 80% ethanol between trials to eliminate olfactory cues. On Day 5, a probe trial was conducted to assess long-term memory retention, during which the escape tunnel was removed and the target hole blocked. Each mouse was allowed to explore the maze for 3 minutes, and time spent in the target quadrant as well as latency to sniff the target hole were recorded to evaluate spatial memory. On Day 15, a remote probe trial was performed under the same conditions as the Day 5 probe, where time spent in the target quadrant and frequency of visits to the target hole were recorded. All measurements were tracked and analyzed using Ethovision XT (Noldus Instruments, Version 15.0) Wageningen, the Netherlands.

### Proteomics sample preparation

Frozen hippocampal tissue samples were lysed and homogenized in lysis buffer (4% SDS, 100 mM Tris-HCl, 1 mM DTT), followed by heating at 85°C for 10 minutes, sonication, and centrifugation to collect the lysate. Protein reduction was performed by adding tris (2-carboxyethyl) phosphine to a final concentration of 5 mM and incubating at 60°C for 20 minutes. Reduced disulfide bonds were alkylated by adding iodoacetamide to a final concentration of 20 mM and incubating for 10 minutes in the dark at room temperature. The single-pot, solid-phase, sample preparation strategy 16 was used to clean up samples for mass spectrometry from solubilized lysates. Proteins were captured onto carboxylate-modified magnetic SpeedBeads (Thermo Fisher Scientific, USA) in a 50% ethanol environment by incubating at 24°C for 5 minutes with shaking (900 rpm). Using a magnetic rack, beads were isolated and the supernatant discarded. The beads were washed three times with 80% ethanol, each time discarding the supernatant. Trypsin protease was added at a 1:20 (w/w enzyme:protein) ratio in 20 mM ammonium bicarbonate and incubated overnight at 37°C with shaking. The peptide solution was then separated from the beads and collected into fresh tubes. Digested samples were acidified with trifluoroacetic acid to 0.5% (v/v) before desalting using the StageTip method 17 and drying using a speedvac. Due to the large number of samples, tissues were processed in three separate batches for mass spectrometry analysis.

### LC-MS analysis of peptides

Liquid chromatography-mass spectrometry (LC-MS) was performed using a Thermo Ultimate 3000 RSLCnano UHPLC system coupled to a Thermo Orbitrap Eclipse Tribrid mass spectrometer (Thermo Fisher Scientific, USA). Peptides were reconstituted in 0.1% (v/v) trifluoroacetic acid (TFA) and 2% (v/v) acetonitrile (ACN), and 500 ng of peptides were loaded onto a PepMap C18 5 µm 1 cm trapping cartridge (Thermo-Fisher Scientific, USA) at 12 µL/min for 6 minutes, before switching the trap in-line with the analytical column (nanoEase M/Z Peptide BEH C18 Column, 1.7 µm, 130 Å, 75 µm ID × 25 cm, Waters). The column compartment was maintained at 55°C throughout the analysis. Peptide separation was performed at 250 nL/min using a linear ACN gradient with buffer A (0.1% (v/v) formic acid, 2% (v/v) ACN) and buffer B (0.1% (v/v) formic acid, 80% (v/v) ACN), starting at 14% buffer B, increasing to 35% over 90 minutes, then rising to 50% B over 15 minutes, followed by 95% B in 5 minutes. The column was cleaned for 5 minutes at 95% B, then ramped down to 1% B over 2 minutes, and held at 1% B for a final 3 minutes. Mass spectra were acquired using the Thermo Orbitrap Eclipse in Data Independent Acquisition (DIA) mode, with MS1 ("master scan") spectra and HCD DIA spectra collected. MS1 scan parameters included a scan range of 350-1400 m/z, 120,000 resolution, maximum injection time of 50 ms, and automatic gain control (AGC) target of 1e6 (250%). DIA spectra were collected with a 3-second target cycle time, 30K resolution, a 15 m/z isolation window, and 45 windows covering a precursor range of 361-1036 m/z, with a scan range of 197-2000 m/z. The AGC target for DIA spectra was set to 1e6 (2000%), with a maximum injection time of 55 ms and an HCD collision energy of 30%. Internal mass correction was applied using the Thermo EASY-IC method.

### Database search

The mass-spectral database search was performed using DIA-NN v1.8.1 18 with label-free DIA matching against the Mus musculus reference proteome downloaded from UniProt (UP000000589_10090, one protein sequence per gene, March 2023). A spectral library was generated through deep learning in DIA-NN using the FASTA file, and library precursors were reannotated from the FASTA database. The fragment size range was set to 200-1800 m/z, with methionine excision enabled. The peptide length range was 7-30 amino acids, while the precursor m/z range was 360-1030, with a precursor charge range of 1-4. Trypsin/P was used for digestion, and the number of allowed missed cleavages was set to 2. Fixed peptide modifications included carbamidomethylation of cysteine, while variable modifications included acetylation of the protein N-terminus and oxidation of methionine, with up to three variable modifications per peptide permitted. The following processing parameters were applied: "use quant," "peptidoforms," "reanalyse," "relaxed protein inference," and "smart profiling." Mass tolerances were automatically learned from the dataset, and the false discovery rate (FDR) threshold for protein validation was set to 0.01.

### Proteomics data analysis

A combination of normalization strategies, statistical tests, and machine learning-based feature selection was employed to systematically identify Differentially Expressed Proteins (DEPs) between experimental groups. First, missing values were imputed by replacing them with the minimum observed value of the corresponding protein (column-wise) minus a small constant (0.05), preserving the data structure while minimizing artificial bias. To correct for systematic technical variation, three distinct normalization techniques were applied separately: mean normalization, which centered each sample's intensity values around the overall mean to adjust for baseline differences; ratio normalization, which scaled intensities relative to a reference sample or group mean to account for proportional disparities; and binomial transformation, which converted continuous expression values into categorical data (e.g., overexpressed vs. underexpressed). Following quality control, proteomics data from batches 1 and 3 were combined for downstream analysis, as batch 2 exhibited significant technical artifacts. This multi-strategy normalization ensured robust feature selection across diverse data distributions.

### Signature discovery by attribute weighting models

An ensemble learning approach integrating multiple attribute weighting (feature selection) models was employed to identify differentially expressed proteins (DEPs). Feature selection was conducted using RapidMiner Studio (version 9.10, RapidMiner GmbH, Dortmund, Germany). Ten distinct attribute weighting algorithms were applied to independently assess each protein's predictive power for group classification, including entropy-based models (Information Gain, Information Gain Ratio), statistical models (Chi-Squared, Deviation), rule-based models (Rule, Gini Index), a distance-based model (Relief), a Support Vector Machine (SVM)-based model, the Uncertainty Coefficient, and Principal Component Analysis (PCA). For datasets processed with binomial normalization, only seven attribute weighting algorithms were utilized. Each model assigned a binary weight (1 = selected, 0 = not selected) based on predefined thresholds, and proteins were ranked according to their cumulative score across all ten models. The top 200 proteins with the highest total scores were selected for further analysis, ensuring stability across different selection algorithms 19, 20.

### Recursive feature elimination (RFE) for feature refinement

To refine the feature set and remove redundant or noisy variables, Recursive Feature Elimination (RFE) was implemented using custom Python scripts within the Anaconda Distribution environment (Anaconda Inc., Austin, TX, USA). Feature ranking was conducted using three distinct machine learning models: Logistic Regression (LR), which ranked features based on the absolute magnitude of model coefficients; Random Forest (RF), which utilized Mean Decrease in Impurity (Gini importance) to assess feature significance; and Linear Support Vector Machine (SVM), which ranked features according to the magnitude of their coefficients in the hyperplane equation. The RFE process involved iteratively eliminating the lowest-ranked features, retraining the model on the reduced feature set at each step, and determining the optimal subset based on performance metrics such as accuracy or cross-validation scores 19, 21. Finally, the intersection and union of features selected by these models were used to construct a robust subset for further analyses.

### Statistical validation using Mann-Whitney U test

To assess differential expression between groups, the non-parametric Mann-Whitney U test was used for its robustness in analyzing non-normally distributed proteomic data and comparing two independent groups. The analysis was conducted in R software (version 4.4.0) using the wilcox.test function, with a significance threshold set at p < 0.05. After identifying proteins through attribute weighting, RFE, and the Mann-Whitney U test, a second Mann-Whitney U test was performed on the merged feature list to validate statistical significance. Only proteins that met the significance threshold (p < 0.05) at this stage were retained for pathway analysis and visualization.

### Heatmap visualization and pathway enrichment analysis

To assess the biological relevance of the identified protein biomarkers, protein expression levels were first standardized using z-score normalization (mean = 0, standard deviation = 1) across samples. These standardized values were then visualized as heatmaps in GraphPad Prism (version 10), facilitating clear interpretation of expression patterns and clustering across experimental groups. Protein-protein interaction networks were analyzed using STRING (version 12). Gene set enrichment analysis (GSEA, version 4.3.3) was conducted on the proteomic dataset using the desktop application with curated mouse GO biological processes and pathway databases (version 2023.2). Next, the biological significance of differentially expressed proteins (DEPs) was examined using Ingenuity Pathway Analysis (IPA; Qiagen), which mapped the proteins to canonical pathways, biological functions, and interaction networks. Significant pathways and networks were identified using IPA's right-tailed Fisher's exact test, with a significance threshold of p < 0.05.

### Statistical analysis

**Figure 1:**
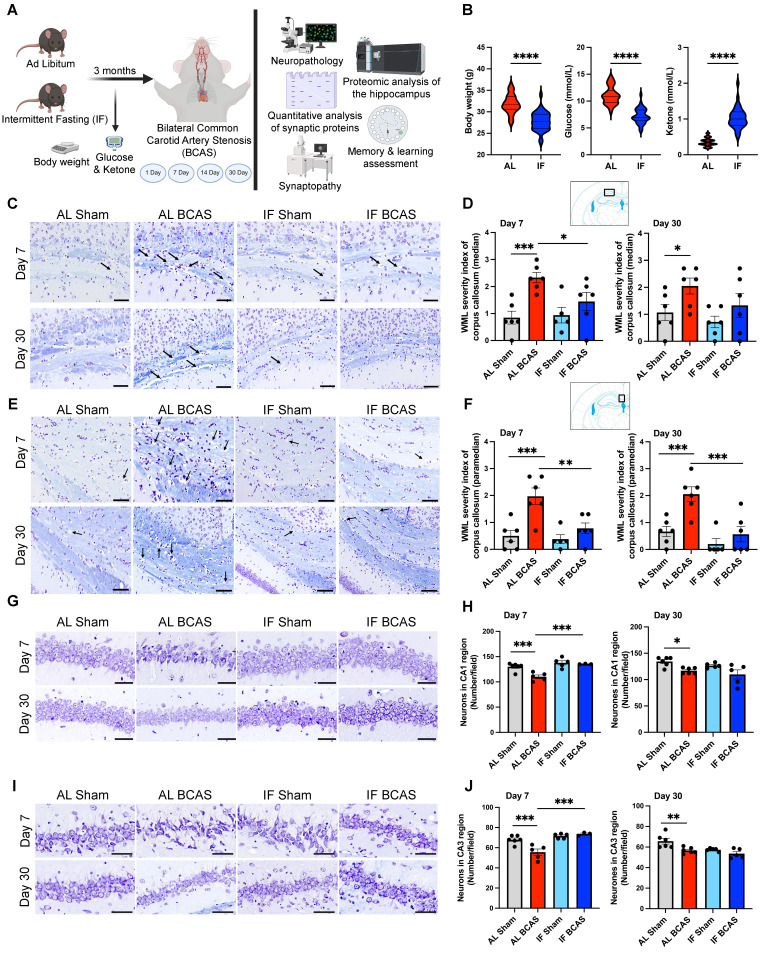
** Intermittent fasting (IF) promotes metabolic switching and attenuates neuropathological alterations induced by chronic cerebral hypoperfusion (CCH).** (A) Schematic outline of experimental groups, timeline, and design. Three-month-old male C57BL/6 mice were assigned to ad libitum (AL) or intermittent fasting (IF) groups; the IF group underwent daily 16-hour fasting for three months. At six months, both groups underwent either bilateral common carotid artery stenosis (BCAS) or sham surgery. Assessments included Barnes maze testing, neuropathology, synaptic protein quantification, synaptic structure and density analysis, and quantitative proteomics analysis of hippocampus. Analyses were performed at days 1, 7, 14, and 30 post-BCAS. Body weight and blood glucose and ketone levels were measured at experimental endpoint (B) Violin plots illustrate the physiological effect of IF compared to AL feeding. At the experimental endpoint, IF mice exhibited significantly lower body weight, reduced blood glucose levels, and elevated blood ketone levels relative to AL mice. n = 30-36 mice in each experimental group. ****P < 0.0001. (C and D) and (E and F) show representative luxol fast blue-stained sections and corresponding quantitative analyses of white matter lesions in the median (C and D) and paramedian (E and F) regions of the corpus callosum at the 7 and 30-day timepoints of the AL- CCH, IF-CCH and the respective sham mice. Scale bar: 50 μm. Lesions were characterized by myelin rarefaction and structural disruption. White matter disruption was graded: Grade 0 = no disruption; Grade 1 = disarranged nerve fibers; Grade 2 = marked vacuole formation; Grade 3 = loss of myelinated fibers. A clear reduction in WML severity was observed in IF mice compared to AL mice following CCH. (G and H) and (I and J) Representative crystal violet-stained images and quantification illustrating the loss of Nissl-positive neurons in the CA1 and CA3 regions of the hippocampus at days 7 and 30 post-CCH in AL mice. Scale bar: 100 μm. No significant differences were observed between IF-CCH and IF-sham groups. Notably, at day 7 post-CCH, IF-CCH mice exhibited a significantly higher number of neurons in CA1 and CA3 regions compared to AL-CCH mice (H and J). Data are represented as mean ± S.E.M. n = 5-6 mice in each experimental group. *P < 0.05; **P < 0.01; ***P < 0.001.

**Figure 2:**
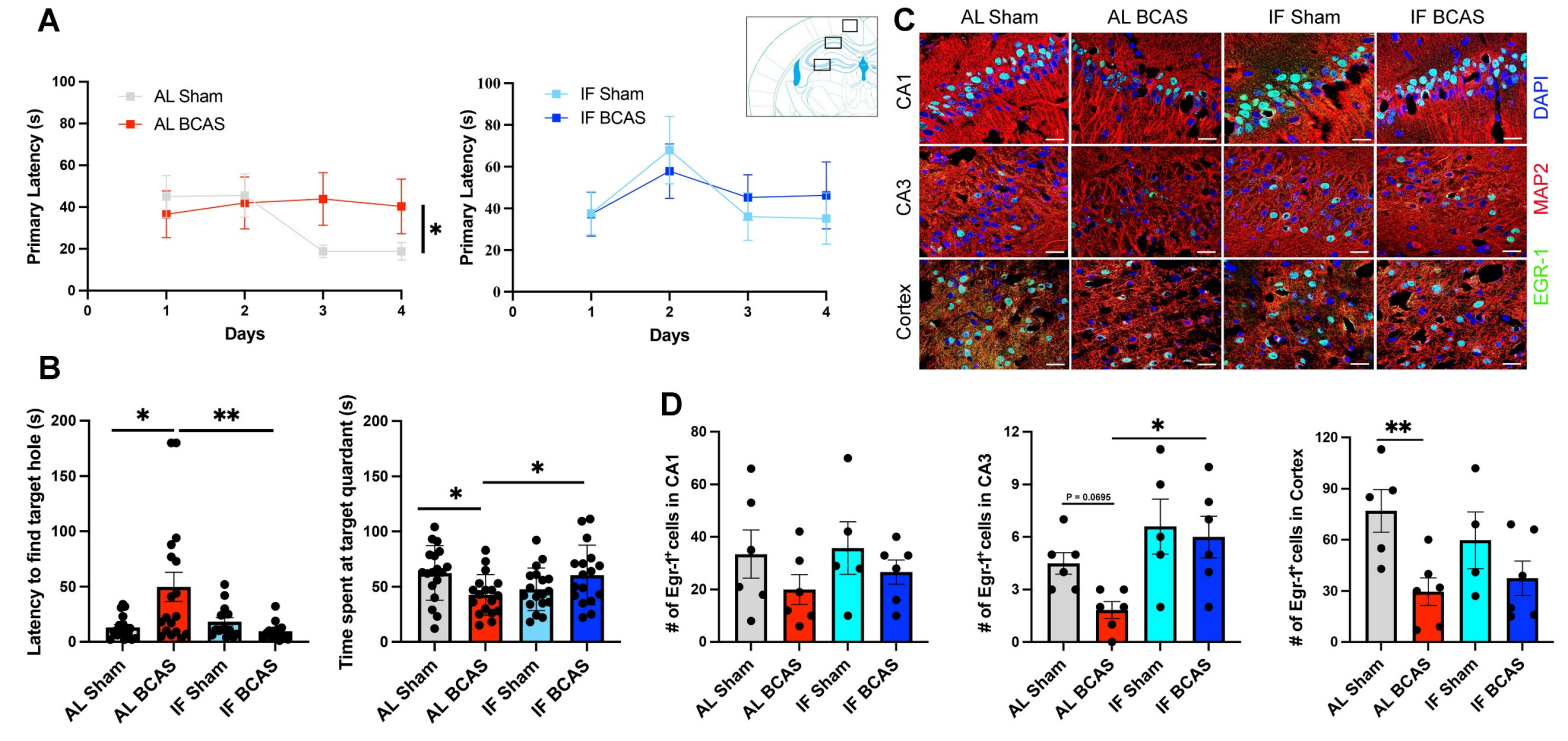
**Intermittent fasting (IF) mitigates chronic cerebral hypoperfusion (CCH) induced cognitive deficits.** (A and B) Barnes maze testing was conducted on AL and IF mice subjected to CCH. Learning ability was evaluated by measuring the latency to enter the target hole during the acquisition phase; AL-CCH mice exhibited significantly longer latencies compared to AL-sham controls on days 3 and 4 (A). No significant differences were observed between IF-CCH and IF-sham groups (A). Spatial memory retrieval was assessed on Day 15 using a reprobe test. AL-CCH mice demonstrated increased latencies (B) and reduced time spent in the target quadrant relative to AL-sham mice (B), indicative of memory impairment. In contrast, IF-CCH mice showed significantly reduced latencies (B) and increased time spent in the target quadrant compared to AL-CCH mice (B), reflecting improved spatial memory retention. Data represented as mean ± S.E.M. n = 13-18 mice per experimental group. (C-D) IF preserves Early Growth Response Protein 1 (EGR-1) expression in chronic cerebral hypoperfusion. Immunohistochemical analysis shows reduced EGR-1 expression in AL BCAS mice compared to IF BCAS mice in hippocampal CA3 and cortical regions following reprobe test. Scale bar: 20 μm. (D) Quantitative analysis reveals significantly higher EGR-1-positive cell counts in IF BCAS mice versus AL BCAS controls in the CA3 region. Data represent mean ± S.E.M. n = 5-6 mice in each experimental group. *p < 0.05; **P < 0.01.

**Figure 3:**
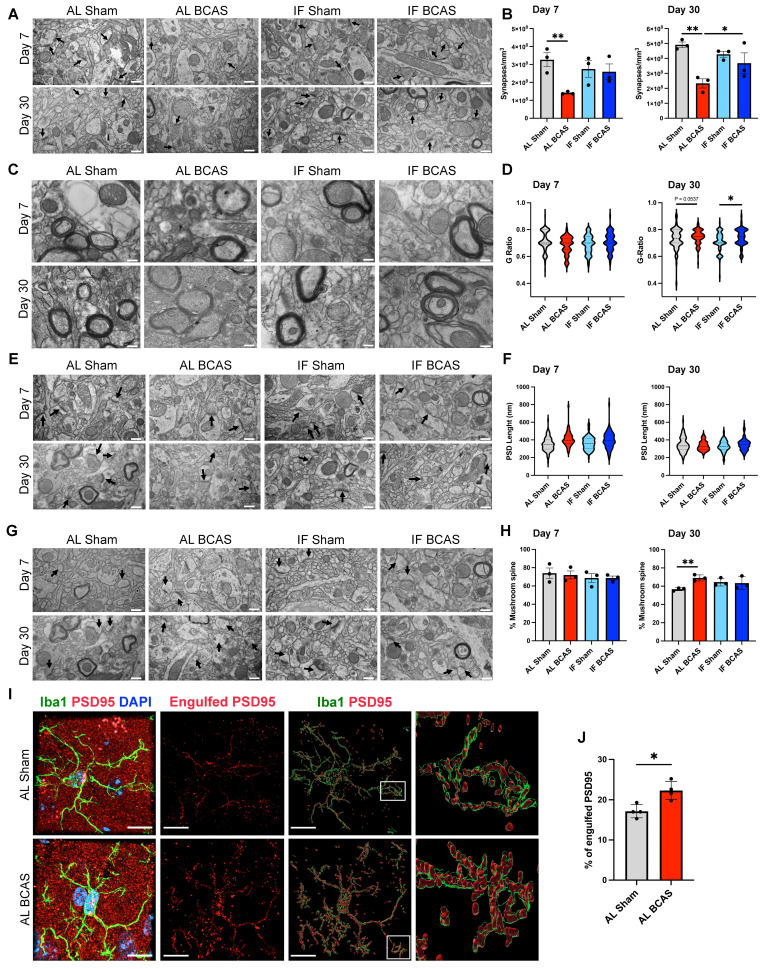
** Assessment of the modulatory effects of intermittent fasting on chronic cerebral hypoperfusion (CCH)-induced synaptic loss and ultrastructural alteration.** (A and B) Representative transmission electron microscopy (TEM) images (A) and corresponding quantification (B) demonstrated significant decrease in synaptic density in AL-CCH mice in prefrontal cortex region at both day 7 and day 30 post-CCH, with no significant differences observed between IF-CCH and sham groups. (C and D) Ultrastructural analysis of the g-ratio indicated thinner cortical myelin sheaths (reflected by a higher g-ratio) in both AL-CCH and IF-CCH mice at day 30 compared to control (D). (E and F) Representative TEM images and quantification showed a marked increase in postsynaptic density (PSD) length (did not reach significant level) in both AL-CCH and IF-CCH groups only at day 7 post CCH compared to sham controls. (G and H) A significant increase in the number of mushroom-shaped spine in AL-CCH mice at day 30 post-CCH, with no difference observed between IF-CCH and sham. TEM images were taken at 10000 magnification, scale bar: 600 nm. (I and J) Representative IMARIS images and quantification illustrate a significant upregulation in microglial engulfment of the postsynaptic protein PSD95 in AL-CCH mice at day 7 post-CCH. Scale bar: 5 µm. Data are represented as mean ± S.E.M. n = 3-4 mice in each experimental group. *P < 0.05, **P < 0.01.

**Figure 4:**
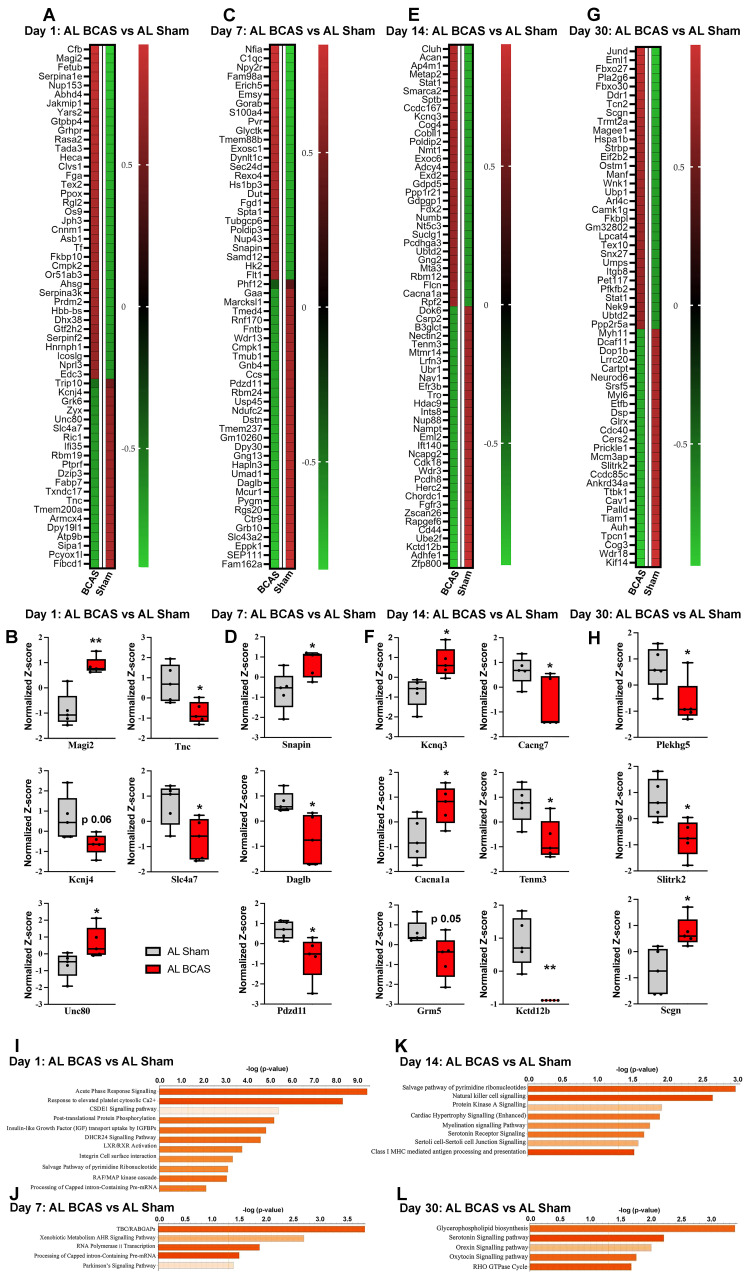
** Temporal proteomic profiling of hippocampal tissue reveals synaptic alterations and pathway changes following chromic cerebral hypoperfusion (CCH).** Heatmaps showing differentially expressed proteins in response to CCH (BCAS) at day 1 (A), day 7 (C), day 14 (E), and day 30 (G), with colours representing z-score normalized protein abundances (red: higher abundance; green: lower abundance). Boxplots illustrating changes in key proteins involved in synaptic transmission at, day 1 (B), day 7 (D), day 14 (F), and day 30 (H) in response to CCH compared the control sham. Pathway enrichment and molecular network analyses were conducted using Ingenuity Pathway Analysis (IPA). (I-L) Bar plots are showing significantly enriched canonical pathways in response to CCH at different time points. The Y-axis represents -log(p-value), and each bar corresponds to a pathway, with colour indicating predicted activation state based on the z-score, orange indicates predicted activation (positive z-score). Longer bars indicate greater statistical significance, with pathways ranked by significance. n = 5 mice in each experimental group. *P < 0.05, **P < 0.01.

All statistical analyses were performed using GraphPad Prism version 10.0. Data are presented as means ± standard error of the mean (SEM) unless otherwise specified. Welch's t-test was used for Figure 1B. For multi-group comparisons in Figures 1D, F, H, and J, we first conducted one-way ANOVA followed by Fisher's LSD post hoc test to identify specific pairwise differences between group means. For the behavioral testing data in Figure 2A, a two-way repeated-measures ANOVA was conducted, followed by Bonferroni's multiple comparisons test to determine p-values relative to control groups. The one-way ANOVA with Fisher's LSD test was used for Figures 2B and D to identify specific pairwise differences. In Figures 3B and H, statistical significance was assessed using a one-way ANOVA with Fisher's LSD test, whereas Figures 3D and F (plotted as nested data and visualized with violin plots) were analyzed using a nested one-way ANOVA. Welch's t-test was applied to Figure 3J and Figures 4, 6 and S7, and all data in Figures S1 and S2 were evaluated using a one-way ANOVA with Fisher's LSD test.

## Results and Discussion

The study design includes the timing of experimental interventions, behavioral analyses, and tissue collections (Figure 1A). To assess the impact of a 16-hour IF regimen on energy metabolism, we monitored body weight, blood glucose levels, and ketone concentrations over the four-month dietary intervention period. IF animals demonstrated significantly lower body weight compared to ad libitum (AL) mice (Figure 1B), consistent with prior reports that IF promotes metabolic efficiency through weight management and enhanced lipid utilization 22. Notably, IF mice exhibited reduced blood glucose and elevated ketone levels (Figure 1B), suggesting a metabolic shift toward ketogenesis, which may confer neuroprotection by providing alternative energy substrates and reducing oxidative stress 23.

### Intermittent fasting mitigates white matter lesions, neuronal cell death, and cognitive decline induced by chronic cerebral hypoperfusion

To evaluate IF's protective effects against VaD pathologies induced by CCH, we examined white matter (WM) integrity and neuronal loss. WM disruption was most pronounced in the corpus callosum of AL BCAS animals at 7 and 30 days post-CCH (Figure 1C-F), a hallmark of CCH-induced axonal degeneration and demyelination 1. Strikingly, IF BCAS animals showed no significant WM rarefaction compared to IF Sham controls (Figure 1D-F), implying that IF preserves WM integrity. The WM severity index in the internal capsule was elevated in AL BCAS mice at 30 days compared to AL Sham mice, but it remained unchanged in IF BCAS mice compared to IF Sham mice (Figure S1A-B), further supporting IF's role in mitigating microvascular injury. In contrast, no significant differences in WM severity index were detected between groups in the caudoputamen (Figure S1C-D) (Figure S1C-D). BCAS-induced neuronal loss in the CA1 and CA3 regions (Figure 1G-J) aligns with prior evidence that CCH exacerbates hippocampal vulnerability due to glutamate excitotoxicity and impaired energy supply 2. However, IF BCAS animals exhibited near-normal neuronal density and protected from CCH-induced neuronal loss (Figure 1H-J), suggesting that IF enhances neuronal resilience 7, 8.

Cognitive assessments via the Barnes Maze revealed that AL BCAS animals had impaired learning and long-term memory (Figure 2A-B), consistent with hippocampal and WM damage disrupting spatial navigation circuits 24. In contrast, IF BCAS animals performed comparably to IF Sham controls (Figure 2B), highlighting IF's ability to maintain synaptic plasticity and memory consolidation despite CCH. The reduced latency and prolonged target-quadrant preference in the Barnes Maze for IF BCAS mice during the remote probe test (Figure 2B) may reflect preserved synaptic function. We have previously showed that IF mice exhibit significantly improved learning ability, including reduced latency to enter the target quadrant during both the probe and remote probe tests 8. However, in this cohort we did not observe improved memory during the probe test (Figure S2A-B). This divergence may arise from differences in environmental cues between cohorts, affecting stress-dependent memory retrieval. The preserved remote memory (despite unimproved initial probe performance) implies that IF's protective effects may emerge selectively during memory consolidation, possibly through delayed reductions in neuroinflammation or enhanced late-phase synaptic stabilization. Finally, the reduction in early growth response protein 1 (EGR-1) expression in AL BCAS animals (Figure 2C-D) indicates impaired activity-dependent synaptic plasticity, a key mechanism in VaD 25. EGR-1 plays a critical role in synaptic plasticity, learning, memory, and neuronal adaptation to stress 26. The normalization of EGR-1 in IF BCAS mice implies that IF restores neuronal responsiveness to environmental cues, possibly via intermittent metabolic switching-driven epigenetic modulation 27.

### Intermittent fasting preserves synaptic structural integrity against chronic cerebral hypoperfusion-induced loss

To investigate the potential mechanistic role of synaptic integrity in IF-mediated protection against cognitive decline, we analyzed the expression levels of key pre- and post-synaptic proteins, including Syntaxin 6, Syntaxin 1A, Synapsin, Synaptophysin, Neuroligin, PSD95, and Shank (Figure S3). Immunoblot analysis was performed on brain tissue samples collected at Days 1, 7, 14, 21, and 30 following BCAS surgery. Surprisingly, no significant differences in synaptic protein expression were observed between Sham-operated and BCAS animals at any time point (Figure S3A-B), suggesting that CCH-induced cognitive decline may occur independently of gross synaptic protein loss. This contrasts with prior reports of synaptic protein downregulation in acute ischemia 28 but aligns with studies showing functional synaptic deficits preceding molecular changes in chronic hypoperfusion models 29, 30, 31. To further validate these findings, we conducted quantitative hippocampal proteomic analysis of synaptic protein levels on Day 7 and Day 30 post-surgery. Consistent with the immunoblot data, proteomic analysis revealed no significant alterations in the expression of pre- or post-synaptic markers between the Sham and BCAS groups (Figure S3C). To independently validate these findings, we performed IHC analysis of synaptic markers in brain sections from Sham and BCAS animals at Day 7 and Day 30. Consistent with immunoblot and proteomic data, IHC revealed no detectable changes in staining intensity or spatial distribution of pre- and post-synaptic proteins between experimental groups (Figure S4A-D). Together, these results from orthogonal methodologies (immunoblotting, proteomics, and IHC) robustly demonstrate that BCAS-induced cognitive decline is not associated with gross perturbations in synaptic protein expression, suggesting that IF's protective effects may involve alternative mechanisms such as synaptic functional modulation, metabolic adaptation, or non-cell-autonomous pathways.

To examine synaptic ultrastructural alterations induced by CCH, we performed quantitative electron microscopy analysis of synaptic density on dendritic spines within a 50-100 µm radius from the mid-cingulate cortical cell layer. Our analysis revealed a significant synaptic loss in BCAS animals compared to Sham controls at both 7 and 30 days post-surgery, demonstrating progressive synapse degeneration under hypoperfusion (Figure 3A-B). Notably, IF seemingly prevented this BCAS-induced synaptic depletion, with IF BCAS animals maintaining synapse densities equivalent to Sham controls. Importantly, no differences were observed between AL Sham and IF Sham groups (Figure 3B), indicating that IF itself does not alter baseline synaptic architecture. These findings demonstrate that while chronic hypoperfusion induces rapid and sustained synaptic degeneration, IF provides remarkable preservation of synaptic connectivity in the cerebral cortex, suggesting its potential to maintain functional neural circuits despite reduced cerebral blood flow. We next examined whether CCH alters axonal myelination by calculating g-ratios (inner axon diameter/total fiber diameter) in the cerebral cortex following BCAS (Figure 3C-D). While 7-day CCH showed no significant g-ratio differences between Sham and BCAS groups, 30-day CCH induced g-ratio increases in both AL and IF BCAS groups compared to respective Sham groups, indicating late-onset myelin thinning. To assess potential synaptic reorganization under CCH, we examined post-synaptic density (PSD) length, a structural indicator of excitatory synapse size and molecular complexity. Our ultrastructural analysis revealed distinct temporal patterns in PSD remodeling following BCAS (Figure 3E-F). At the 7- and 30- days timepoints, both AL-fed and IF groups showed no change in PSD lengths compared to their respective Sham controls (Figure 3F). Notably, IF did not appear to modify this response pattern at either timepoint examined. Evaluation of dendritic spine subtypes showed no significant differences in mushroom spine density between the Sham and BCAS groups under either dietary condition at 7 days post-BCAS. However, by 30 days post-BCAS, the AL BCAS group exhibited a significant increase in mushroom spine density compared to AL Sham controls (Figure 3G-H). Mushroom spines are characterized by their large synaptic heads and narrow necks—are critical for maintaining stable, long-term synaptic connections and are typically associated with established memory circuits. Given the observed synaptic loss following BCAS, we investigated whether CCH-induced synapse elimination involves microglia-mediated engulfment. Using super -resolution confocal microscopy, we detected significantly increased PSD95^+^ synaptic material internalized within Iba1^+^ microglial cells in the cortical region following 7-days AL BCAS (Figure 3I-J) implicating neuroinflammation in synaptic loss. IF's ability to preserve synapses may reflect upstream suppression of "eat-me" signals (e.g., reduced complement C3 tagging) 32 or enhanced neuronal resistance to detachment.

### Proteomic profiling of the hippocampus reveals key disease pathways and protein alterations in chronic cerebral hypoperfusion

To elucidate key pathobiological processes impacting synaptic integrity in the hippocampus following chronic cerebral hypoperfusion (CCH), we conducted quantitative proteomic profiling at 1, 7, 14, and 30 days post-bilateral common carotid artery stenosis (BCAS) (Fig. 3A-L). Our analysis revealed temporally dynamic alterations in protein expression and disease-associated pathways linked to synaptic dysfunction. At 1-day post-BCAS, we identified significant dysregulation of synaptic proteins compared to Sham-operated animals (Figure 4A). Notably, our proteomic analysis revealed significant dysregulation of key synaptic regulators at 1-day post-BCAS (Figure 4B). Membrane-associated guanylate kinase inverted-2 (Magi2), a postsynaptic scaffolding protein essential for maintaining glutamatergic receptor clustering 33 and synaptic stability, and Unc80 (Unc-80 Homolog), a critical component of the NALCN channel complex that modulates neuronal resting membrane potential 34, were both significantly upregulated (Figure 4B). Conversely, we observed downregulation of Tnc (tenascin-C), an extracellular matrix glycoprotein involved in synaptic plasticity 35; Slc7a7 (L-type amino acid transporter 1), which mediates lysosomal nutrient sensing 36; and Kcnj4 (inwardly rectifying potassium channel Kir2.3), a regulator of neuronal excitability 37 (Figure 4B). Analysis 7-days post- BCAS demonstrated distinct hippocampal proteomic signatures (Figure 4C). Three synaptic regulators exhibited significant time-dependent alterations: SNAPIN (synaptosomal-associated protein interacting protein), a vesicle docking protein critical for synaptic vesicle recycling 38, showed marked upregulation at 7 days post-BCAS. Conversely, we observed significant downregulation of DAGLB (diacylglycerol lipase beta), the primary biosynthetic enzyme for 2-arachidonoylglycerol (2-AG) endocannabinoid signaling 39, and PDZD11 (PDZ domain-containing protein 11), a scaffolding protein implicated in synapse organization 40 (Figure 4D). These findings reveal progressive synaptic proteome reorganization during early CCH pathogenesis.

Extended 14-day CCH induced a distinct hippocampal proteomic signature (Figure 4E), characterized by significant dysregulation of key synaptic regulators. Among upregulated proteins, we identified KCNQ3 (potassium voltage-gated channel subfamily Q member 3), a critical modulator of neuronal M-current that regulates excitability 41, and CACNA1A (calcium voltage-gated channel subunit alpha1 A), the pore-forming subunit of P/Q-type calcium channels essential for synaptic transmission 42. Conversely, four synaptic proteins showed marked downregulation: CACNG7 (calcium voltage-gated channel auxiliary subunit gamma 7), a regulator of AMPA receptor trafficking 43; TENM3 (teneurin-3), an extracellular matrix protein mediating synaptic partner matching 44; GRM5 (metabotropic glutamate receptor 5), a key postsynaptic Gq-coupled receptor modulating synaptic plasticity 45; and KCTD12B (potassium channel tetramerization domain containing 12B), a fast-acting GABA receptor modulator 46 (Figure 4F). Extended 30-day CCH revealed a distinct hippocampal proteomic profile compared to sham-operated controls, with pronounced alterations in synaptic regulators (Figure 4G). Notably, we identified significant downregulation of PLEKHG5 (pleckstrin homology and RhoGEF domain containing G5), a Rac1 activator crucial for spine morphogenesis 47, and SLITRK2 (SLIT and NTRK-like family member 2), a postsynaptic adhesion molecule regulating excitatory synapse development 48. Conversely, SCGN (secretagogin), a calcium-sensor protein implicated in synaptic vesicle exocytosis 49, showed marked upregulation (Figure 4H). These persistent alterations suggest chronic synaptic adaptation mechanisms and reorganization in prolonged CCH.

To elucidate the functional consequences of CCH-induced hippocampal proteomic alterations and their potential links to neuronal cell death and cognitive decline, we performed pathway enrichment and molecular network analyses (Figure 4I-L) and protein-protein interaction network analysis (Figure S5). Our pathway enrichment and molecular network analyses of proteins dysregulated at 1-day post-BCAS identified significant enrichment in several critical pathways (Figure 4I): (1) acute-phase response signaling, reflecting early neuroinflammatory activation; (2) cellular response to elevated cytosolic calcium, indicative of excitotoxic stress 9; (3) post-translational protein modification and insulin-like growth factors, linking to synaptic maintenance mechanisms 50; (4) integrin cell surface interactions, important for blood-brain barrier integrity 51; and (5) RAF/MAP kinase cascade, associated with apoptotic signaling 52 (Figure 4I). To further characterize the temporal progression of CCH-induced molecular disturbances and their association with cognitive impairment, we extended our pathway enrichment and protein-protein interaction analyses to later time points. At day 7 post-BCAS, highly dysregulated pathways included: (1) TBC/RABGAP signaling, implicating vesicular trafficking deficits 53; (2) AHR-mediated transcriptional regulation, suggesting expression of inflammatory cytokines and chemokines genes 54; (3) RNA polymerase II transcription, reflecting transcriptional dysregulation 55; and (4) Parkinson's disease signaling, potentially linking to neurodegenerative processes (Figure 4J). These findings demonstrate a temporal shift from acute stress responses (observed at 1 day) to chronic adaptive and degenerative pathways at 7-days post-BCAS. At 14 days post-BCAS, pathway analysis revealed chronic dysregulation of key processes including: (1) the pyrimidine ribonucleotide salvage pathway, indicating altered nucleotide metabolism and potential DNA repair deficits 56; (2) natural killer cell signaling, reflecting sustained neuroimmune activation 57; (3) PKA signaling, suggesting impaired cAMP-mediated synaptic plasticity 58; (4) myelination signaling, consistent with emerging white matter damage; (5) serotonin receptor signaling, potentially underlying mood-related behavioral changes; (6) cell junction signaling, implicating persistent blood-brain barrier dysfunction; and (7) class I MHC-mediated antigen presentation, demonstrating adaptive immune system engagement 59 (Figure 4K). These late-phase alterations—spanning metabolic adaptation, immune recruitment, and structural reorganization—contrast with acute stress responses, collectively illustrating the temporal evolution of CCH pathophysiology.

Finally, at 30 days post-BCAS, our proteomic analysis identified persistent dysregulation in several critical pathways (Figure 4L): (1) glycerophospholipid biosynthesis, may indicate sustained membrane lipid metabolism disturbances and potential myelin remodeling 60; (2) serotonin receptor signaling, suggesting long-term neuromodulatory dysfunction 61; (3) orexin neuropeptide signaling, potentially linking to sleep-wake cycle disruptions and feeding behavior 62; (4) oxytocin signaling, which may influence social cognitive deficits 63; and (5) RHO GTPase cycle, reflecting ongoing cytoskeletal reorganization and synaptic instability 64. These late-stage alterations demonstrate the progression of CCH pathophysiology from acute stress responses to chronic neuromodulatory and structural adaptations, potentially underlying the persistent cognitive and behavioral deficits observed in chronic cerebral hypoperfusion models. To elucidate functional relationships among dysregulated synaptic proteins, we performed STRING network analysis of temporally up- and down-regulated targets. This revealed three distinct protein-protein interaction (PPI) clusters corresponding to acute (1 day), subacute (7-14 days), and chronic (30 days) phases of CCH (Figure S5).

### Hippocampal proteomic profiling reveals key neuroprotective pathways associated with intermittent fasting

**Figure 5:**
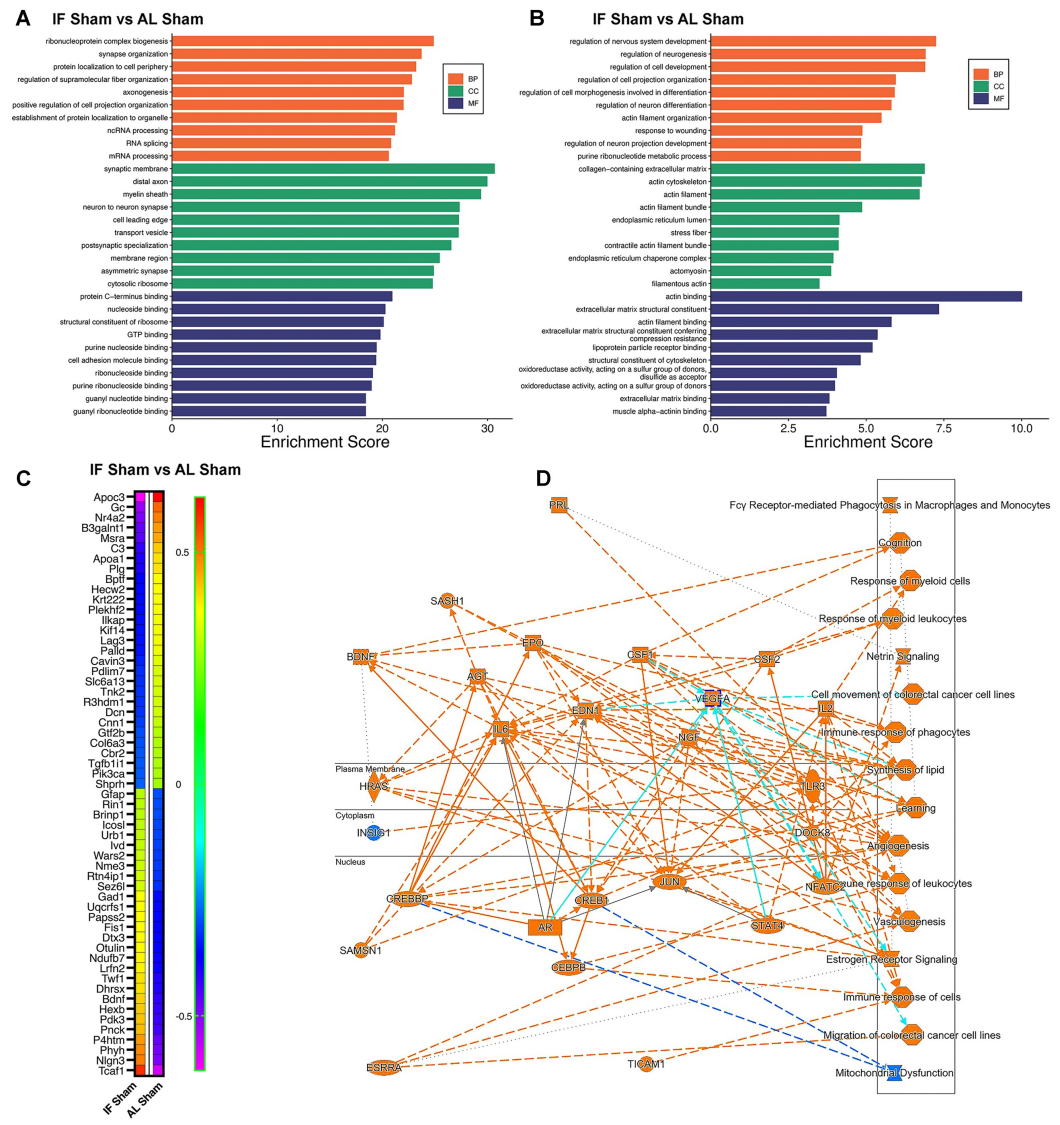
**Intermittent fasting (IF) changes the proteomic landscape of hippocampus**. We first examined the effect of IF on the hippocampus under normal physiological conditions. (A and B) Bar plots showing the top enriched Gene Ontology (GO) terms across three categories—Biological Process (BP), Cellular Component (CC), and Molecular Function (MF)—modulated by IF. Bars represent normalized enrichment scores. Two analyses were performed: (A) using the entire proteomics dataset from AL-sham and IF-sham groups, and (B) using significantly differentially expressed proteins (DEPs). (C) Heatmap displaying DEPs in response to IF compared to AL. (D) Graphical summary of key canonical pathways and biological functions altered by IF, identified using Ingenuity Pathway Analysis (IPA). Predicted pathway activation states are indicated by z-scores (orange: activation; blue: inhibition). n = 20 mice in each experimental group.

We next systematically analyzed if and how IF remodels the hippocampal proteome by performing Gene Ontology (GO) enrichment analysis across three categories: Biological Process (BP), Cellular Component (CC), and Molecular Function (MF) (Figure 5 A-B). Our analysis proceeded in two stages: first examining the entire proteome to identify broad IF-induced changes (Figure 5A), followed by a focused analysis of significantly differentially expressed proteins (DEPs) to reveal specific neuroprotective mechanisms (Figure 5B**)**. Initial whole-proteome analysis comparing IF-Sham and AL-Sham groups demonstrated IF's global impact on hippocampal biology. Biological processes most affected included ribonucleoprotein complex biogenesis, synapse organization, and axonogenesis, indicating IF enhances both structural and functional aspects of neuronal connectivity. Cellular component analysis revealed significant enrichment in synaptic membranes, myelin sheath proteins, and neuron-to-neuron synapses, while molecular functions showing the strongest modulation involved nucleotide binding and protein C-terminus interactions. These whole-proteome findings suggest IF broadly supports synaptic maintenance and RNA processing under basal conditions (Figure 5A**)**. Our subsequent DEP analysis uncovered more specific pathways through which IF may confer neuroprotection. Biological processes regulating nervous system development and neurogenesis were prominently represented, along with pathways involved in neuron differentiation and projection development. At the cellular level, DEPs clustered in extracellular matrix components, cytoskeletal structures, and ER chaperone complexes, while molecular functions centered on extracellular matrix binding and cytoskeletal organization (Figure 5B**)**. These DEP-specific patterns reveal how IF targets fundamental processes of neuronal structure and connectivity, potentially explaining its ability to preserve cognitive function despite cerebrovascular challenges.

Our analysis identified several highly modulated hippocampal proteins in response to IF, as shown in the heatmap (Figure 5C). Among the most significantly downregulated proteins were Apoc3 (implicated in lipid metabolism and neuroinflammation) 65, and complement C3 (a key mediator of neuroinflammation) 66 - reductions that collectively suggest IF attenuates inflammatory pathways while modulating neuroprotective signaling. Conversely, prominently upregulated proteins included synaptic organizer Nlgn3, activates Gαi1/3-Akt signaling to protect neuronal cells from hypoxic injury 67, metabolic regulator Pdk3 (involved in neurotransmitter synthesis and energy metabolism) 68, and P4htm (a hypoxia-responsive enzyme) 69, indicating IF enhances both synaptic integrity and metabolic adaptation. The heatmap additionally reveals numerous other highly modulated proteins involved in diverse neuronal functions, with clustering patterns demonstrating coordinated regulation of protein networks related to synaptic plasticity, oxidative stress response, and cellular homeostasis (Figure 5C). These findings provide a comprehensive molecular signature of IF's effects on the hippocampal proteome, revealing how it simultaneously targets multiple neuroprotective mechanisms including inflammation reduction, synaptic preservation, and metabolic optimization.

Furthermore, Ingenuity Pathway Analysis (IPA) revealed a comprehensive network of canonical pathways and biological functions modulated by IF (Figure S6), as graphically summarized (Figure 5D**)**. The analysis demonstrated significant upregulation of proteins involved in cognition-related pathways, Netrin signaling, and angiogenesis, suggesting IF enhances neurotrophic signaling and vascular remodeling. These upregulated pathways correlate with our behavioral findings of preserved cognitive function and may explain IF's ability to maintain synaptic plasticity through Netrin-1 mediated axon guidance mechanisms 70. Conversely, we observed downregulation of mitochondrial dysfunction pathways, consistent with IF's known role in improving mitochondrial biogenesis and efficiency. The coordinated regulation of these pathways - enhancing neuroplasticity while reducing metabolic stress - provides a systems-level explanation for IF's neuroprotective effects in CCH, particularly its ability to preserve both neuronal function and vascular integrity despite reduced cerebral blood flow. These findings suggest IF acts through multiple complementary mechanisms to create a neuroprotective state resilient to hypoperfusion-induced damage.

### Intermittent fasting modulates the proteomic landscape to safeguard against synaptic loss induced by chronic cerebral hypoperfusion

**Figure 6:**
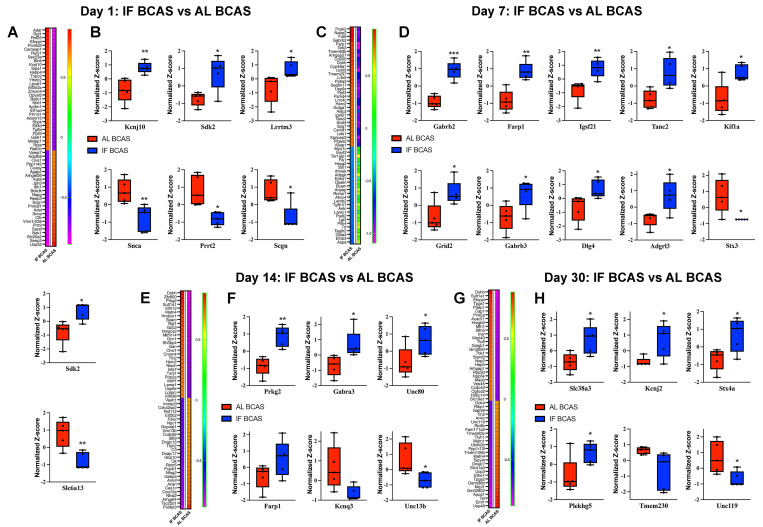
** Proteomic analysis uncovers the molecular mechanisms underlying intermittent fasting (IF) mediated protection against Chronic cerebral hypoperfusion (CCH) induced hippocampal pathology.** Heatmaps of DEPs and boxplots showing expression of key synaptic transmission-related proteins in response to IF at days 1 (A and B), 7 (C and D), 14 (E and F), and 30 (G and H) post-CCH. The impact of IF under CCH conditions by analysing proteomic profiles from IF-CCH and AL-CCH groups. n =5 mice in each experimental group. *P < 0.05, **P < 0.01, ***P < 0.001.

To systematically characterize the temporal evolution of hippocampal proteomic alterations induced by CCH, we performed longitudinal proteomic profiling across acute (1 and 7 days), subacute (14 days), and chronic (30 days) phases following BCAS and investigated how IF modulates these CCH-induced proteomic changes, aiming to identify key molecular pathways underlying IF-mediated neuroprotection. Our temporal analysis of hippocampal proteomic changes revealed distinct protein expression patterns between IF-BCAS and AL-BCAS groups across acute (Day 1 and 7), subacute (Day 14), and chronic (Day 30) phases of cerebral hypoperfusion (Figures 6).

Focusing on synaptic regulators, we identified significant early modifications at Day 1 post-BCAS (Figure 6A-B), with IF-BCAS animals showing elevated expression of Kcnj10 (Kir4.1 potassium channel), Sdk2 (synaptic adhesion molecule), and Lrrtm3 (glutamatergic synapse organizer) compared to AL-BCAS controls. These upregulated proteins collectively enhance synaptic stability through complementary mechanisms: Kcnj10 maintains optimal extracellular potassium levels for neuronal excitability 71), Sdk2 promotes excitatory synapse assembly 72, and Lrrtm3, postsynaptic adhesion molecules essential for excitatory synapse development 73. Concurrently, IF-BCAS animals exhibited reduced levels of Snca (α-synuclein), Prrt2 (stress granule component), and Scgn (calcium sensor), suggesting IF simultaneously attenuates pathological processes including protein aggregation (Snca) 74, regulator of Ca^2+^ sensitivity at glutamatergic synapses (Prrt2) 75, and dysregulated calcium signaling (Scgn) 76. These Day 1 proteomic changes establish an early neuroprotective signature that precedes the structural synaptic preservation we observed ultrastructurally, indicating IF initiates rapid molecular adaptations that may underlie its beneficial effects against hypoperfusion-induced synaptic dysfunction.

At Day 7 post-BCAS, proteomic analysis revealed a distinct synaptic reorganization pattern in IF-treated animals (Figure 6C-D), characterized by significant upregulation of Gabrb2 and Gabrb3 GABA receptor subunits, suggesting enhanced inhibitory tone that may counteract hypoperfusion-induced hyperexcitability 77. The concurrent elevation of synaptic scaffolding proteins Dlg4 (PSD-95) 78 and Adgrl3 (latrophilin-3) 79 indicates strengthened postsynaptic density organization, while increased Farp1 and Tanc2 implicate improved actin cytoskeleton remodeling at dendritic spines 80. Notably, the persistent upregulation of Sdk2 from Day 1 through Day 7 reinforces its potential role in maintaining excitatory synapse integrity during prolonged hypoperfusion. The significant reduction in Stx3, a presynaptic vesicle regulator 81, and Slc6a13, a GABA transporter, suggests IF may modulate neurotransmitter release dynamics while maintaining optimal extracellular GABA levels 82. These coordinated changes demonstrate IF's ability to establish a balanced synaptic environment by Day 7 - enhancing structural stability through scaffold proteins (Dlg4, Tanc2), while fine-tuning neurotransmission via receptor (Gabrb2/3) and transporter (Slc6a13) regulation. The emergence of kinesin Kif1a as a newly upregulated factor at this stage suggests IF may additionally support axonal transport mechanisms, potentially compensating for hypoperfusion-related deficits in vesicle trafficking. This multipronged synaptic reorganization precedes our observed cognitive protection at later time points, suggesting these Day 7 modifications may represent critical early adaptations that enable maintained neural circuit function despite chronic hypoperfusion.

At Day 14 post-BCAS, analysis identified significant hippocampal protein modifications in IF-treated animals that reveal distinct neuroprotective adaptations (Figure 6E-F). The upregulation of Prkg2, a cGMP-dependent kinase, suggests enhanced synaptic plasticity mechanisms 83. Elevated Gabra3 levels indicate strengthened GABAergic inhibition, which may counterbalance hypoperfusion-induced excitotoxicity 84, while increased Unc80 points to maintained neuronal excitability regulation through NALCN cation channel complex 85. The sustained elevation of Farp1 demonstrates ongoing stabilization of dendritic spine architecture via cytoskeletal remodeling pathways 86. Conversely, reduced levels of Kcnq3, a potassium channel regulating neuronal M-currents 87, and Unc13b, a presynaptic vesicle priming protein 88, suggest IF promotes adaptive tuning of both intrinsic excitability and neurotransmitter release dynamics. These coordinated changes reveal IF's ability to establish a comprehensive neuroprotective environment by simultaneously enhancing synaptic plasticity (Prkg2), maintaining inhibitory balance (Gabra3), stabilizing membrane properties (Unc80, Kcnq3), and optimizing presynaptic function (Unc13b). The persistence of these modifications at this subacute stage suggests they represent stable adaptive responses rather than transient changes, providing a molecular foundation for the long-term cognitive preservation observed in IF-treated animals.

Proteomic profiling at the chronic phase (Day 30 post-BCAS) uncovered a unique hippocampal protein signature in IF-treated animals that reflects established neuroprotective mechanisms (Figure 6G-H). The upregulation of Slc38a3, a glutamine transporter essential for neurotransmitter recycling, suggests enhanced maintenance of glutamatergic transmission despite chronic hypoperfusion 89. Elevated Kcnj2 levels point to improved potassium homeostasis through inward-rectifying channels, potentially stabilizing neuronal resting membrane potentials during prolonged metabolic stress 90. Increased Stx4a expression indicates preserved vesicular trafficking mechanisms at synaptic terminals 91, while upregulation of Plekhg5 may involve in neuroprotection 92, demonstrates ongoing structural plasticity in dendritic compartments. Conversely, reduced levels of Tmem230, a synaptic vesicle-associated protein 93, and Unc119, a lipid-anchored protein important for protein trafficking 94, may reflect adaptive downregulation of specific synaptic components in response to chronic hypoperfusion. The persistence of these modifications at this chronic phase suggests they represent established protective adaptations rather than transient responses, providing a molecular basis for the long-term synaptic preservation observed in IF-treated animals. Notably, the specific upregulation of Slc38a3 and Plekhg5 highlights IF's dual action in supporting both functional and structural aspects of synaptic integrity during sustained hypoperfusion, while the modulation of Kcnj2 demonstrates continued maintenance of ionic homeostasis even at late timepoints. These findings collectively demonstrate how IF establishes a comprehensive neuroprotective environment that addresses multiple aspects of neuronal vulnerability during chronic cerebral hypoperfusion.

Our temporal proteomic analysis revealed Serpina1b as a consistently downregulated protein across multiple phases of cerebral hypoperfusion, showing significant reduction at Day 7, Day 14, and Day 30 in IF-treated animals compared to controls (Figure S7). This progressive suppression of Serpina1b, a member of the serine protease inhibitor family 95, suggests IF may modulate neuroinflammatory pathways and extracellular matrix remodeling throughout hypoperfusion progression. The sustained downregulation of Serpina1b implies a potential role in IF-mediated neuroprotection, possibly through attenuation of protease-activated inflammatory cascades that contribute to blood-brain barrier dysfunction and synaptic damage in chronic hypoperfusion models. Finally, to systematically characterize the functional relationships among temporally dysregulated synaptic proteins in response to IF versus AL feeding following BCAS surgery, we performed STRING network analysis of DEPs across sequential timepoints (Figure S8). The analysis identified PPI networks: (1) In the acute phase post-BCAS, IF animals exhibited upregulated ribosomal small subunit (RPS-family) proteins alongside downregulated Serpina1 (α1-antitrypsin), suggesting early translational activation coupled with attenuated acute-phase inflammatory signaling; (2) The subacute phase maintained Serpina1 suppression, potentially reflecting sustained modulation of neuroinflammatory pathways.

**Figure 7:**
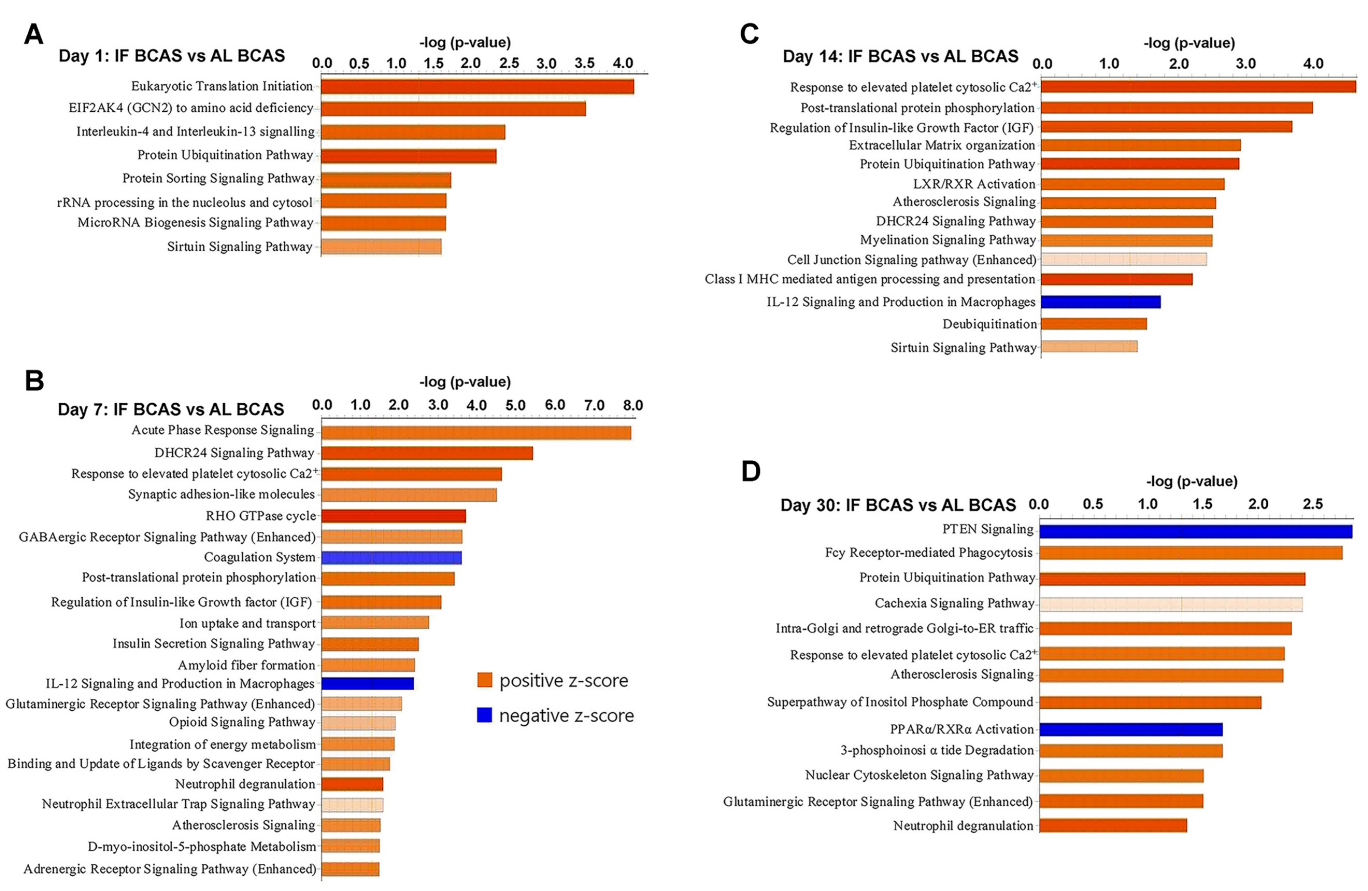
** Proteomic pathways underlying intermittent fasting (IF) mediated protection against Chronic cerebral hypoperfusion (CCH).** Pathway enrichment and molecular network analyses were conducted using Ingenuity Pathway Analysis (IPA). (A-D) Canonical pathway enrichment analysis at indicated time points post-BCAS. Bar plots show significantly enriched pathways with color coding reflecting activation states (orange, activated; blue, inhibited). The Y-axis represents -log(p-value). Data compare IF-CCH versus AL-CCH proteomic profiles. n = 5 mice in each experimental group.

Our temporal pathway analysis revealed distinct neuroprotective mechanisms through which IF modulates the hippocampal response to CCH (Figure 7). The early upregulation of eukaryotic translation initiation and sirtuin pathways at day 1 suggests IF rapidly enhances protein synthesis machinery while activating longevity-associated metabolic regulators - potentially preparing neurons for subsequent hypoperfusion stress through enhanced proteostasis and mitochondrial efficiency (Figure 7A) 97, 98. The concurrent enrichment in interleukin-4/13 signaling indicates a preferential shift toward anti-inflammatory microglial polarization 99, which may create a neuroprotective microenvironment prior to peak ischemic injury. The day 7 profile reveals IF's pleotropic protective strategy, where acute phase response signaling coordinates cellular stress adaptation while synaptic adhesion molecules likely stabilize vulnerable synaptic connections 100. Notably, the suppression of coagulation pathways aligns with emerging evidence that IF improves cerebral microcirculation 7, 101. The sustained upregulation of IGF signaling at both day 7 and 14 (Figure 7B-C) may underlie IF's ability to maintain neuronal survival and plasticity, as IGF-1 is known to enhance neurogenesis and cerebrovascular integrity during metabolic stress 102. By day 14, the emergence of calcium homeostasis and myelination pathways suggests IF promotes adaptive neuronal excitability regulation and white matter preservation - critical factors preventing network hypersynchrony and axonal degeneration in CCH. The persistent protein ubiquitination signature across multiple timepoints implies enhanced clearance of damaged proteins through proteasomal and autophagic pathways 103, while phosphorylation-mediated post-translational modifications may fine-tune synaptic protein function. The late-phase (day 30) dominance of glutamatergic signaling and nuclear-cytoskeletal pathways (Figure 7D) indicates IF facilitates long-term synaptic reorganization, potentially compensating for chronic hypoperfusion through structural plasticity 104. The downregulation of PTEN signaling is particularly significant, as this may release inhibitory constraints on mTOR-mediated protein synthesis and cell survival pathways 105. Conversely, the maintained calcium response signature suggests ongoing adaptation to metabolic stress.

While this study provides novel insights into IF-mediated synaptic preservation in chronic hypoperfusion, several limitations should be acknowledged. First, the exclusive use of male mice precludes assessment of potential sex differences in IF's neuroprotective effects. Second, while our laboratory has previously demonstrated that BCAS induces a consistent 20-30% reduction in cerebral blood flow (CBF) 7, 8, 96, and preliminary measurements confirmed similar hypoperfusion in the current cohort, the lack of systematic longitudinal CBF monitoring in this study prevents the precise temporal correlation between the degree of hypoperfusion and synaptic changes. Third, while proteomics revealed dynamic hippocampal adaptations, mechanistic causality would be strengthened by functional validation through electrophysiology or microglial manipulation. Fourth, synaptic analyses focused primarily on the cortex, potentially overlooking contributions from hippocampal or white matter regions implicated in VaD. Additionally, the fixed 16-hour fasting regimen and use of young adult mice may not fully recapitulate the heterogeneity of human vascular cognitive impairment. While our current findings provide compelling evidence, future studies involving larger cohorts of aged mice are warranted to further validate these results within the context of dementia progression. Additionally, incorporating both sexes, multi-regional assessments, and translational models of aging and small-vessel disease will be crucial in more comprehensively evaluating the therapeutic potential of IF.

## Conclusion

This study provides compelling evidence that IF offers robust neuroprotection against CCH-induced synaptic degeneration and cognitive decline, revealing novel molecular mechanisms that underlie its therapeutic potential in VaD. Using an integrative approach that combines behavioral assessments, ultrastructural imaging, and proteomic analyses, the research demonstrates that IF preserves synaptic density and cognitive function despite sustained hypoperfusion, independent of gross synaptic protein loss. Key findings show that IF maintains synaptic ultrastructure by preventing CCH-induced loss of hippocampal dendritic spines and preserving post-synaptic density dynamics, as evidenced by electron microscopy. It also modulates microglial synaptic pruning, reducing the engulfment of synaptic material by activated microglia and suggesting a dampening of neuroinflammatory synaptic stripping. Furthermore, IF induces a neuroprotective hippocampal proteome marked by upregulation of synaptic stability factors (e.g., *Kcnj10*, *Sdk2*, *Lrrtm3*), enhanced inhibitory tone through *Gabrb2/3*, and suppression of pro-inflammatory mediators such as *Serpina1b* and *C3*. Temporal proteomic profiling reveals that IF supports early synaptic reinforcement, mid-phase suppression of neuroinflammation, and late-stage metabolic and structural stabilization, highlighting its pleiotropic effects across the course of VaD pathogenesis. Notably, the preservation of synapses despite unchanged levels of synaptic proteins suggests that IF safeguards synaptic function through mechanisms such as microglial modulation, enhanced neuronal resilience, or metabolic reprogramming, rather than simply maintaining protein abundance. Future research should investigate the translational potential of IF in clinical settings and further elucidate the complex interactions between metabolic shifts, neuroinflammation, and synaptic integrity. This work advances our understanding of VaD's synaptic pathophysiology and offers a feasible, multi-targeted strategy to combat vascular cognitive impairment.

## Supplementary Material

Supplementary figures.
