# Supplementary material for: Intermittent fasting reprograms the brain proteome to prevent synaptic degeneration and cognitive impairment in vascular dementia
Source: Theranostics. 2025 Jul 25;15(16):8429–50. doi: 10.7150/thno.119422 (PMC12374579; doi:10.7150/thno.119422)
Supplement: Supplementary file 1 — Supplementary figures. [file thnov15p8429s1.pdf]

Supplementary Information

Intermittent fasting reprograms the brain proteome to prevent synaptic degeneration and cognitive impairment in vascular dementia

Nishat I. Tabassum[1,2,*], Sharmelee Selvaraji[3,4], Yibo Fan[1,2] , Vernise Lim[1,2], Xiangru Cheng[1,2], Xiangyuan Peng[1,2], Aayushi Arora[1,2], Vismitha Rajeev[5], Julian Ratcliffe[6], Chad J. Johnson[6], Keshava K. Datta[7], Rohan Lowe[7], Mansour Ebrahimi[1,2], Quynh Nhu Dinh[1,2], T Michael De Silva[1,2,8], Christopher G. Sobey[1,2,8,9], Peiyan Wong[10], Eddie Feng-Ju Weng[11,12], Dong-Gyu Jo[13], Christopher P. Chen[5], Mitchell K.P. Lai[5], Thiruma V. Arumugam[1,2,13,*]

[1] Department of Microbiology, Anatomy, Physiology and Pharmacology, School of Agriculture, Biomedicine and Environment, La Trobe University, Melbourne, Australia.
[2] La Trobe Institute for Molecular Science, La Trobe University, Melbourne, Australia.
[3] Research Laboratory of Electronics, Department of Materials Science and Engineering, Massachusetts Institute of Technology, Cambridge, MA, USA.
[4] McGovern Institute for Brain Research, Massachusetts Institute of Technology, Cambridge, MA, USA.
[5] Memory Aging and Cognition Centre, Department of Pharmacology, Yong Loo Lin School of Medicine, National University of Singapore, Singapore.
[6] Bioimaging Platform, La Trobe University, Bundoora, VIC, Australia.
[7] La Trobe University-Proteomics and Metabolomics Platform (LTU-PMP), La Trobe Institute for Molecular Science, La Trobe University, Melbourne, Australia.
[8] Centre for Cardiovascular Biology and Disease Research, La Trobe Institute for Molecular Sciences, La Trobe University, Bundoora, Victoria, 3086, Australia.
[9] Baker Heart and Diabetes Institute, Melbourne, Victoria, 3004, Australia
[10] Signature Research Programme in Neuroscience & Behavioural Disorders, Duke-NUS Medical School, Singapore.
[11] Neuroscience and Brain Disease Centre, China Medical University, Taichung, Taiwan.
[12] Graduate Institute of Biomedical Sciences, College of Medicine, China Medical University, Taichung, Taiwan.
[13] School of Pharmacy, Sungkyunkwan University, Suwon, Republic of Korea.

Corresponding authors: Thiruma V. Arumugam or Nishat I. Tabassum, La Trobe Institute for Molecular Science and Department of Microbiology, Anatomy, Physiology and Pharmacology, School of Agriculture, Biomedicine and Environment, La Trobe University, Bundoora, VIC, Australia. **E-mail:** g.arumugam@latrobe.edu.au; garriearumugam@gmail.com or N.Tabassum@latrobe.edu.au

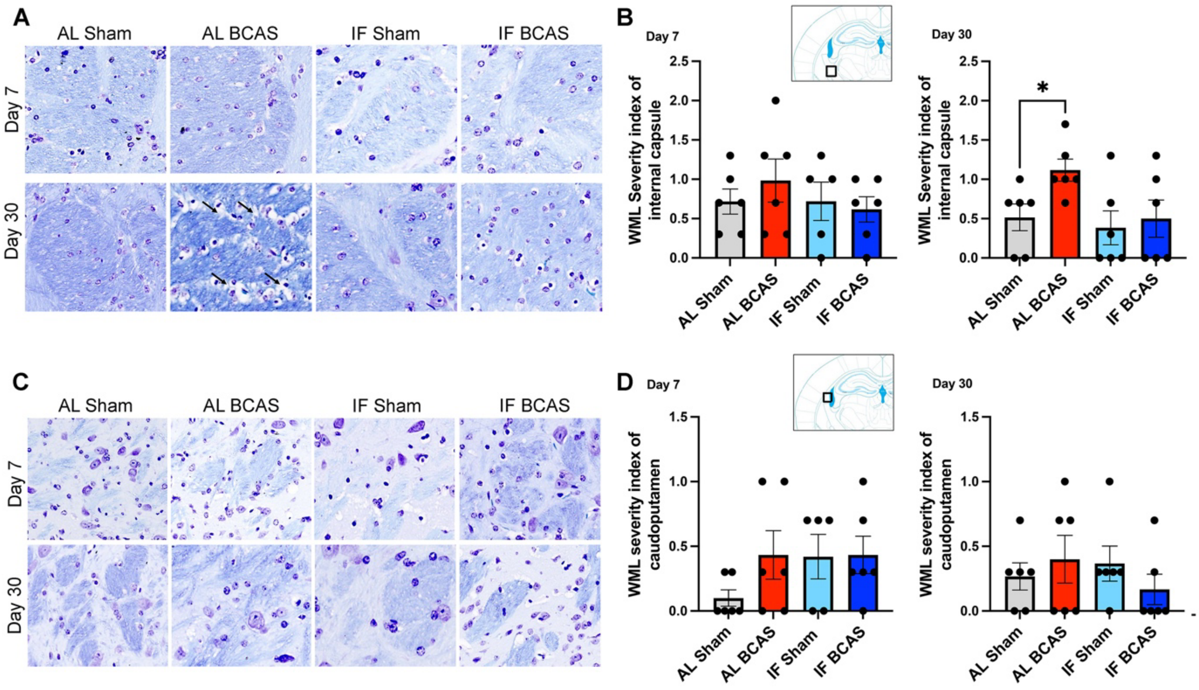

Figure S1: Effect of Intermittent fasting (IF) on chronic cerebral hypoperfusion (CCH) induced white matter lesion (WML). (A and B) Representative Luxol Fast Blue-stained sections and corresponding quantitative analysis showing that, at day 30 post-CCH, AL-CCH mice exhibited significantly increased white matter lesions (WML) in the internal capsule compared to AL-sham controls. In contrast, no significant differences were observed between IF-sham and IF-CCH groups, indicating a protective effect of intermittent fasting. (C and D) The caudoputamen region was also assessed, with no significant differences detected among groups. White matter disruption was graded as follows: Grade 0 = no disruption; Grade 1 = disarranged nerve fibers; Grade 2 = marked vacuole formation; Grade 3 = loss of myelinated fibers. Data are presented as mean ± S.E.M. n = 5-6 mice in each experimental group *$P < 0.05$.

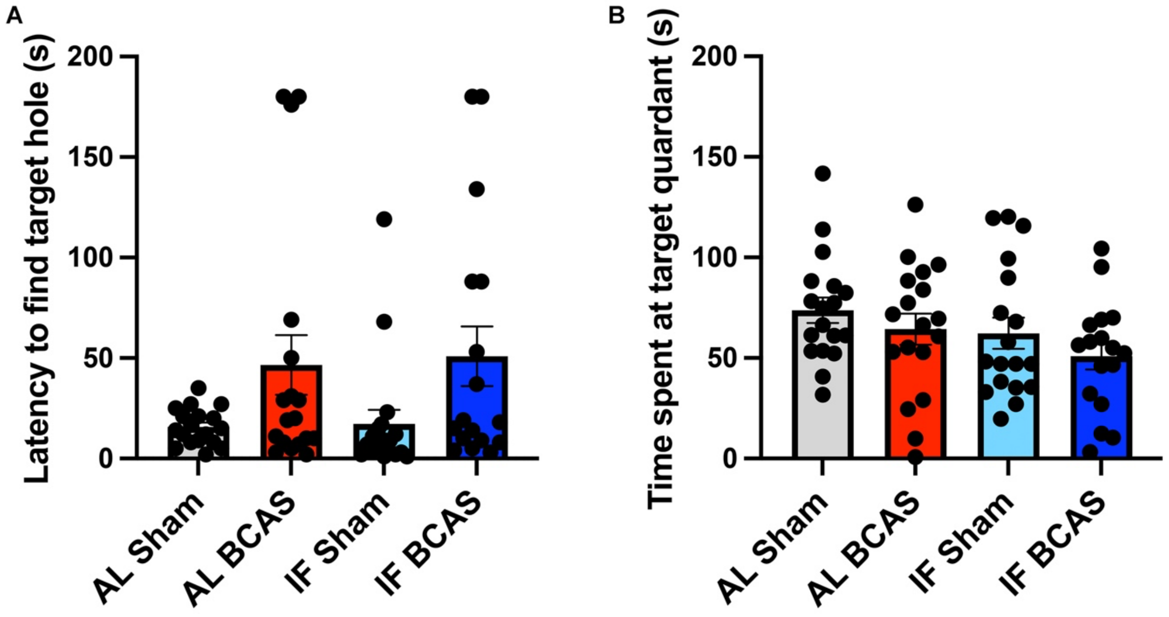

Figure S2: No significant differences in spatial memory retention among experimental groups. On the fifth day following Barnes maze training, a probe test was conducted to evaluate spatial memory retention. Memory retention was assessed by measuring (A) the latency to locate the target hole and (B) the time spent in the target quadrant. No significant differences were observed between groups. Data are presented as mean ± S.E.M. n = 13-18 mice in each experimental group.

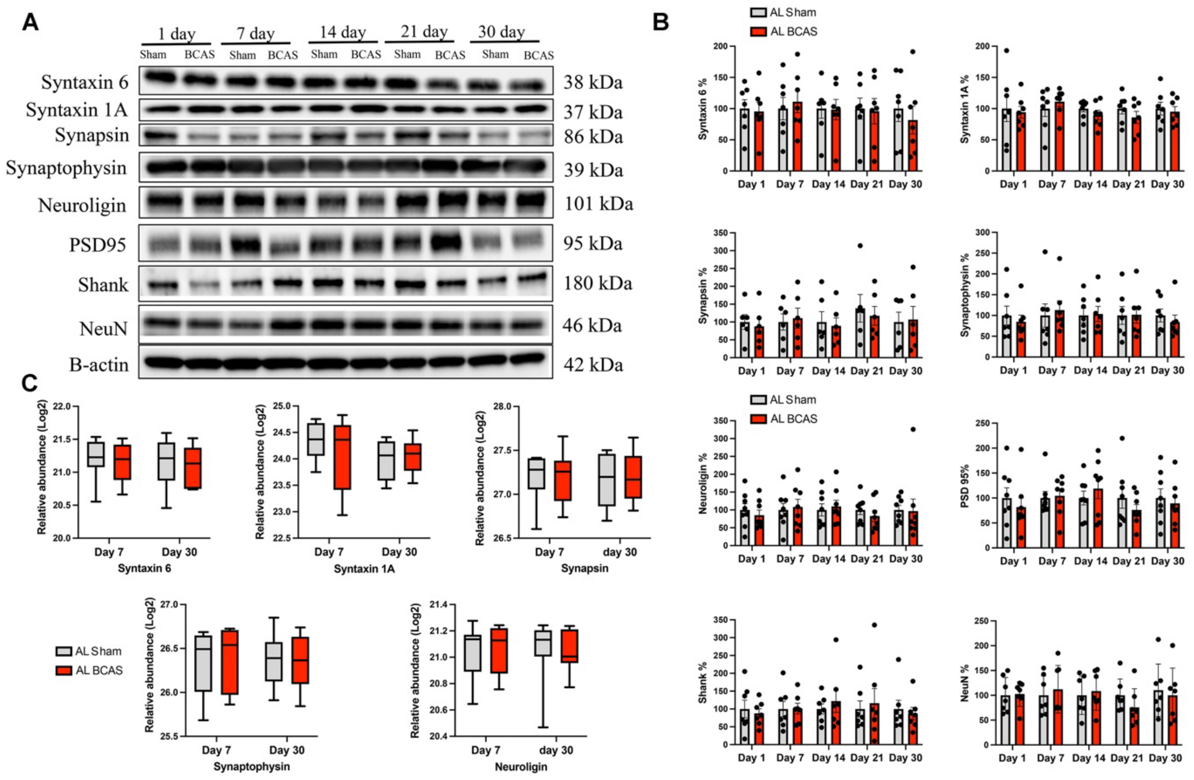

Figure S3: Expression levels of key pre- and post-synaptic proteins remained stable following Chronic cerebral hypoperfusion (CCH) in cortex and hippocampus. (A and B) Representative Western blots and corresponding quantitative analysis show no significant differences in the total protein levels of the nuclear marker NeuN, as well as key pre- and post-synaptic proteins in the cortex, between AL-CCH and sham groups up to 30 days post-CCH. β-actin was used as a loading control. (C) Boxplots display the normalized relative abundance of the same synaptic proteins in hippocampal tissue, based on quantitative proteomics analysis. No significant differences were observed between groups. Data are presented as mean ± S.E.M. n = 5–8 mice in each experimental group.

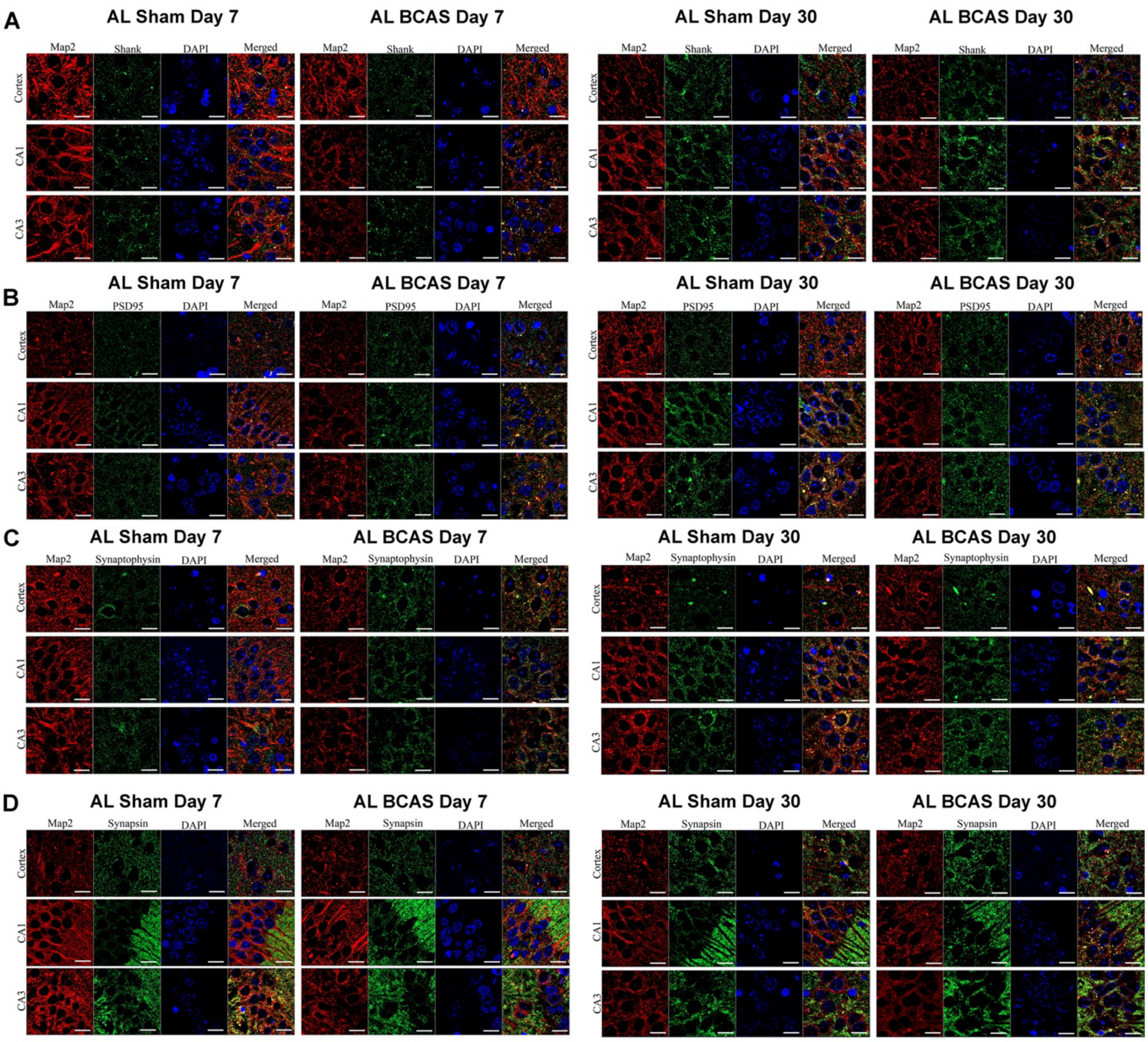

Figure S4: Expression of key pre- and post-synaptic proteins in the cortex and hippocampus following chronic cerebral hypoperfusion (CCH). Representative individual and merged super-resolution immunofluorescence images of the post-synaptic proteins Shank (A) and PSD-95 (B), and the pre-synaptic proteins synaptophysin (C) and synapsin (D), show comparable levels of positive staining between AL-CCH and Sham groups at 7- and 30-days post-CCH in the cortex, CA1, and CA3 regions of the hippocampus. Scale bar = 5 µm. n = 6 mice in each experimental group.

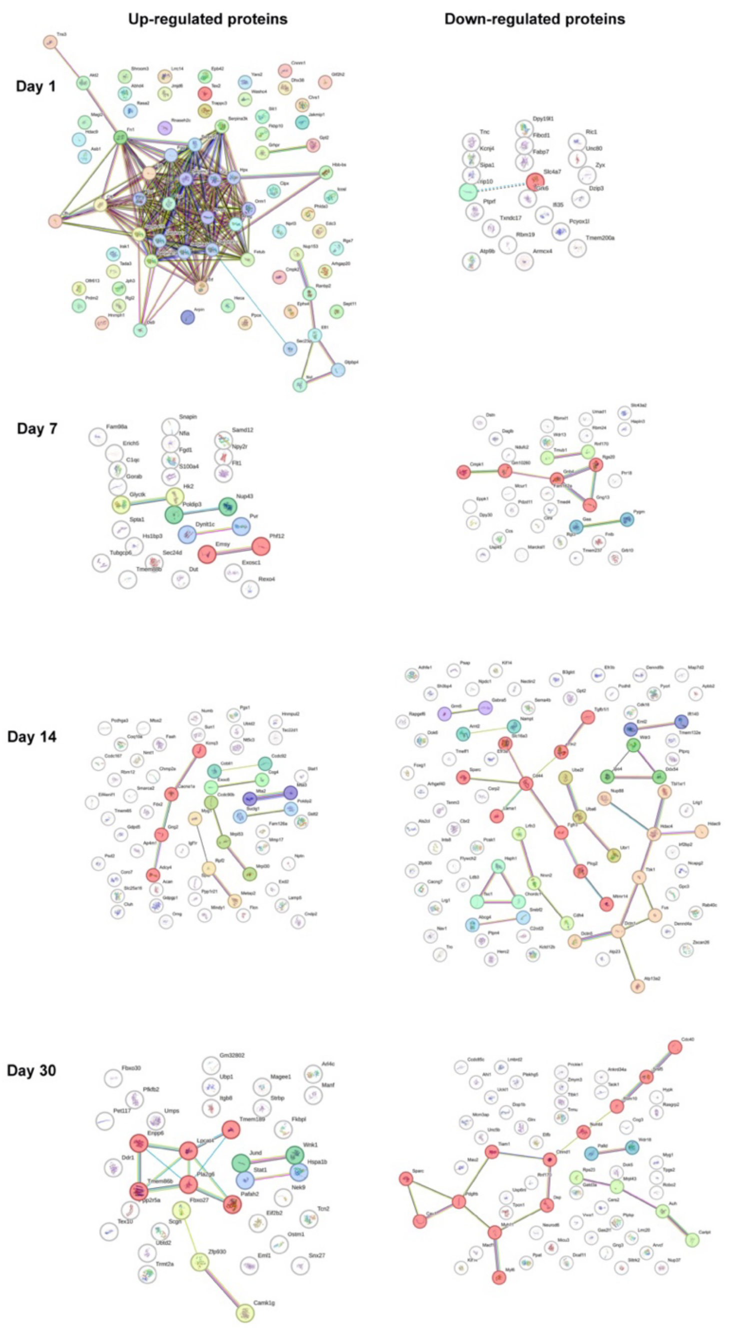

Figure S5: Protein-protein interaction (PPI) networks of differentially expressed proteins in response to chronic cerebral hypoperfusion (CCH). PPI networks were generated using the STRING database for proteins that were upregulated and downregulated in response to CCH compared to sham at each individual time point. Each node represents a protein, while edges indicate predicted functional associations based on known and predicted interactions. Edge colours represent different sources of supporting evidence.

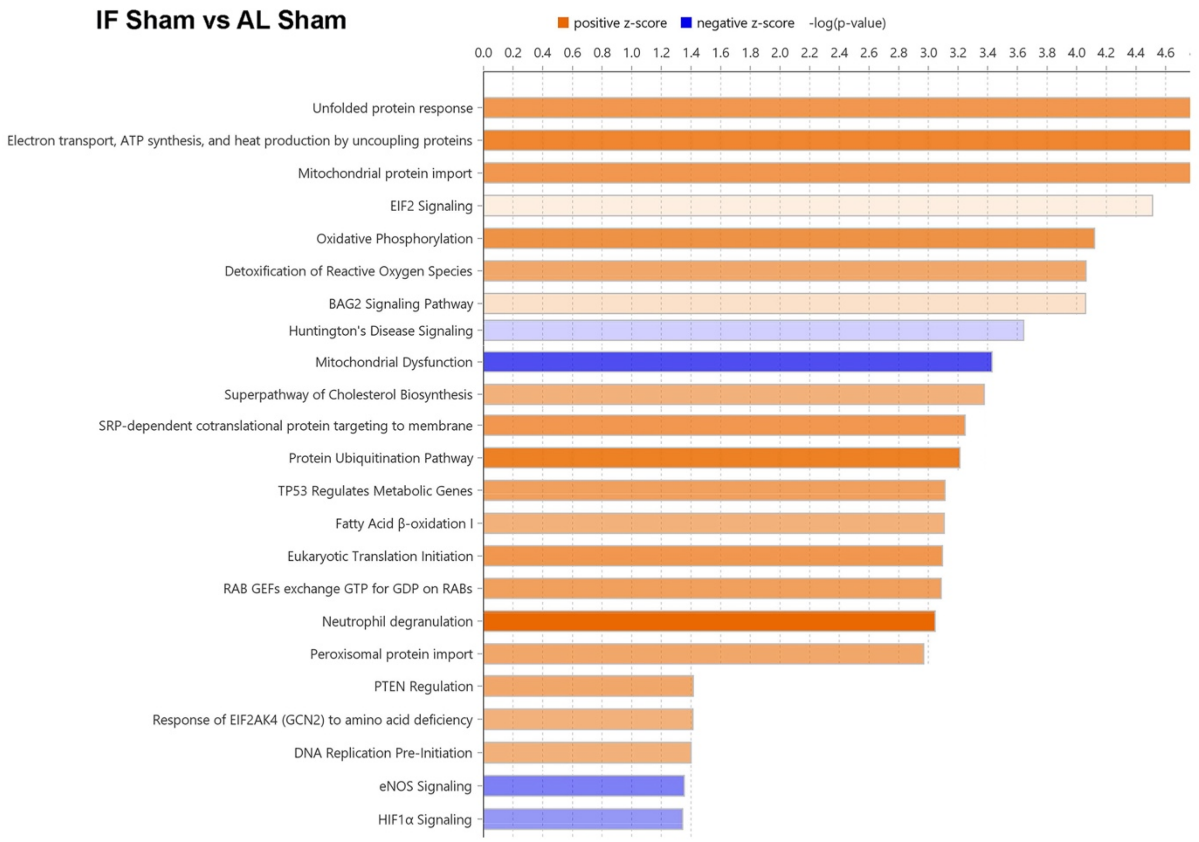

Figure S6: Differentially regulated biological pathways in response to intermittent fasting (IF). IPA was employed to identify enriched canonical pathways in response to IF compared to ad libitum (AL) feeding. The bar graphs represent the top pathways enriched in the dataset in response to IF compared to AL. Bar length corresponds to –log(p-value), indicating the strength of enrichment. Predicted activation states are represented by colour: orange for activated pathways and blue for inhibited pathways, based on z-score.

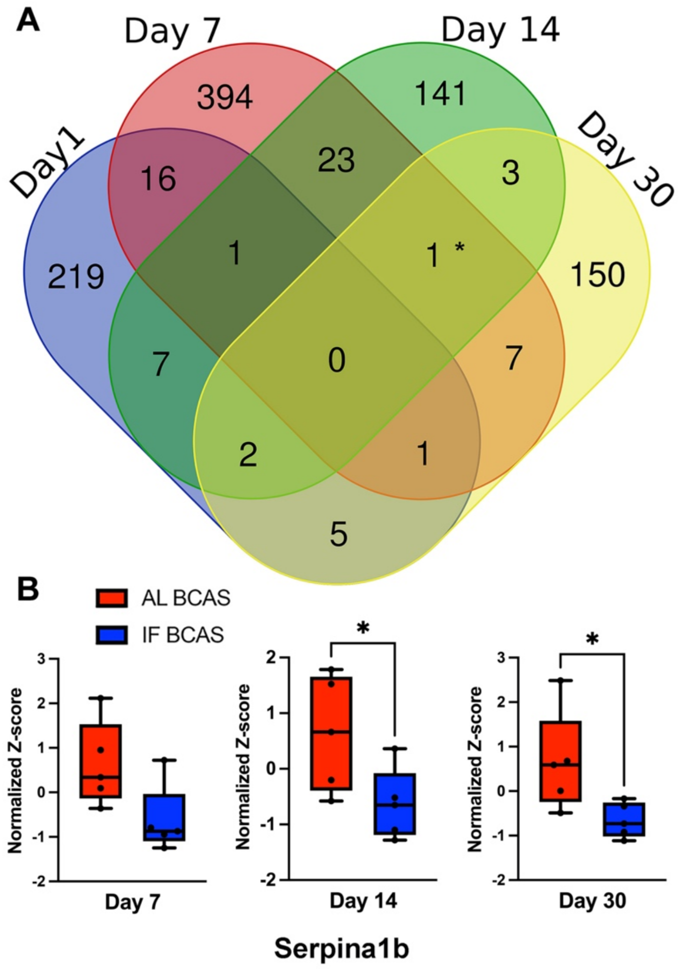

Figure S7: Identification of common proteins across different time points in intermittently fasted (IF) and ad libitum (AL) fed mice following chronic cerebral hypoperfusion (CCH). (A) Venn diagram illustrating the distribution of differentially expressed proteins (DEPs) in AL-CCH and IF-CCH groups across multiple time points. Serpina1b was identified as a unique protein consistently present at days 7, 14, and 30. Serpina1b has been associated with neuroinflammation. (B) Boxplots showing that Serpina1b expression was consistently downregulated in the IF-CCH group compared to the AL-CCH group across these time points. Data are represented as mean ± S.E.M. n = 5 mice in each experimental group. *P < 0.05.

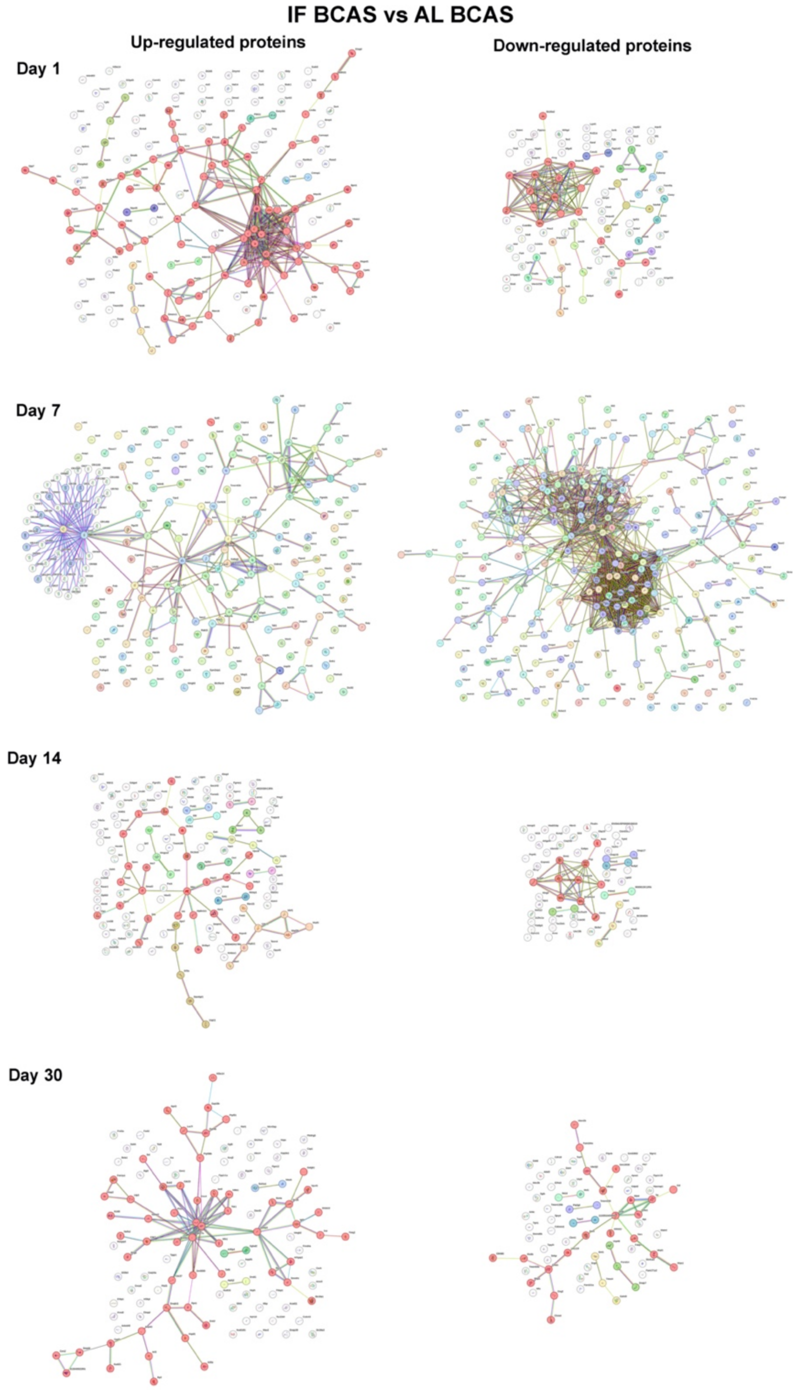

Figure S8: Protein–protein interaction (PPI) networks of differentially expressed proteins in response to intermittent fasting (IF) under chronic cerebral hypoperfusion (CCH). PPI networks were generated using the STRING database, with upregulated and downregulated proteins analysed separately at each time point. Each node represents a protein, and each edge indicates a predicted functional association. Edge colours denote different types of supporting evidence.
